# Ensuring generalizability and clinical utility in mental health care applications: Robust artificial intelligence‐based treatment predictions in diverse psychosis populations

**DOI:** 10.1111/pcn.13914

**Published:** 2025-11-06

**Authors:** Fiona Coutts, Sergio Mena, Esin Ucur, W Wolfgang Fleischhacker, Rene Kahn, Jeffrey Lieberman, Alkomiet Hasan, Oliver Howes, Christoph Correll, Nikolaos Koutsouleris, Paris Alexandros Lalousis

**Affiliations:** ^1^ Institute of Psychiatry, Psychology and Neuroscience, King's College London London UK; ^2^ Department of Psychiatry, Psychotherapy, Psychosomatics and Medical Psychology Medical University of Innsbruck Innsbruck Austria; ^3^ Department of Psychiatry Icahn School of Medicine at Mount Sinai New York New York USA; ^4^ Department of Psychiatry New York State Psychiatric Institute Columbia New York USA; ^5^ University College of Physicians and Surgeons New York City New York USA; ^6^ Department of Psychiatry and Psychotherapy Klinikum der Universität München, Ludwig‐ Maximilians‐University Munich Germany; ^7^ Department of Psychiatry, Psychotherapy and Psychosomatics, Medical Faculty University of Augsburg, BKH Augsburg Augsburg Germany; ^8^ German Center for Mental Health (DZPG), Partner Site Munich—Augsburg Augsburg Germany; ^9^ Department of Child and Adolescent Psychiatry Charité Universitätsmedizin Berlin Germany; ^10^ Center for Psychiatric Neuroscience, Feinstein Institute for Medical Research Manhasset New York USA; ^11^ Department of Psychiatry and Molecular Medicine Zucker School of Medicine at Hofstra/ Northwell Hempstead New York USA; ^12^ German Center for Child and Adolescent Health (DZKJ), Partner Site Berlin Berlin Germany; ^13^ German Center for Mental Health (DZPG), Partner Site Berlin Berlin Germany; ^14^ Max Planck Institute of Psychiatry Munich Germany

**Keywords:** AI, antipsychotics, psychosis, translational, treatment response

## Abstract

**Aim:**

Artificial Intelligence (AI)‐based prediction models of treatment response promise to revolutionize psychiatric care by enabling personalized treatment, but very few have been thoroughly tested in different samples or compared to current clinical standards. Here we present models predicting antipsychotic response and assess their clinical utility in a robust methodological framework.

**Methods:**

Machine learning models were trained and cross‐validated on clinical and sociodemographic data from 594 individuals with established schizophrenia (NCT00014001) and 323 individuals with first episode psychosis (NCT03510325). Models predicted four measures of antipsychotic response at 3 months after baseline. Clinical utility was assessed using decision curve and calibration curve analyses. Model performance was tested in a reduced feature space and across sex, ethnicity, antipsychotic, and symptom change subgroups to investigate model fairness.

**Results:**

Models predicting total symptom severity (*r* = 0.4–0.68) and symptomatic remission (BAC = 62.4%–69%) performed well in both samples and externally validated successfully in the opposing cohort (*r* = 0.4–0.5, BAC = 63.5%–65.7%). Performance remained significant when the models were reduced to 8–9 key variables (*r* = 0.53 for total symptom severity, BAC = 65.3% for symptomatic remission). Models predicting symptomatic remission had a net benefit across risk thresholds of 0.5–0.9 and were moderately well‐calibrated (ECE = 0.16–0.18). Model performance different across sex, ethnicity and medication subgroups.

**Conclusions:**

We present a robust framework for training and assessing the clinical utility of prediction models in psychiatry. Our models generalize across different psychosis populations and show promising calibration and net benefit. However, performance disparities across demographic and treatment subgroups highlight the need for more diverse clinical samples to ensure equitable prediction.

Heterogeneity in treatment response is a common feature in many diseases and a pervasive challenge for medical decision‐making.^1^, ^2^, ^3^ This is particularly the case for psychiatric disorders where patients with the same diagnosis vary in terms of their illness severity, response to medication and risk of relapse.^4^, ^5^, ^6^ Despite this, clinicians have no established predictors to guide medication selection for psychiatric disorders and, consequently, treatment proceeds by trial and error. Crucially, this leads to time and resources lost until the correct treatment is found, resulting in poor clinical and functional outcomes and disease chronicity in roughly every third person.^7^

Precision psychiatry is an approach that aims to tailor treatment of psychiatric disorders to each patient using their unique disease signature by combining data from genetic, imaging, behavioral and/or environmental domains.^8^ As with precision medicine in other fields such as oncology, radiology and cardiology^9^, ^10^, ^11^ artificial intelligence (AI) methods have the potential to transfer this approach from bench to bedside by estimating patients' response likelihood at the individual level prior to treatment start and thus inform clinicians' and patients' decisions. In the case of antipsychotic resistance, for example, a clinician might choose to initiate clozapine treatment earlier for those with a high likelihood of non‐response to conventional antipsychotics, as this is the only effective medication in treatment‐resistant psychosis.^12^ Although many prediction models show promise, particularly in psychosis research,^13^ none have yet been transferred to the clinical setting.

A key reason for this lack of clinical translation is that in order for prediction models to be clinically useful they must robustly generalize their prediction performance across several unseen samples to ensure they are effective in different settings, population, and conditions.^14^ However, a recent review of clinical prediction models in psychiatry found that the majority have not been tested in external data and that 94.5% of these models had a high risk of bias.^15^ Definitions of remission, response, and symptom severity changes following antipsychotic treatment onset vary across studies, further limiting the generalizability of prediction models.^16^ Furthermore, two recently published articles have demonstrated a lack of model generalizability in psychosis populations, which has raised critical doubts on the implementation of precision medicine in psychiatry.^17^, ^18^

The aim of our research was therefore to use rigorous state‐of‐the‐art machine learning methodology to develop clinical AI models to predict changes in psychotic symptom severity following treatment with first‐line antipsychotic medications in two different psychosis populations, and to objectively assess their generalizability across the two samples. These datasets selected for this analysis were chosen because they were diagnostically aligned but differed significantly in terms of illness stage and geographical location, thus providing a robust and conservative testbed to test our models' generalizability. Since definitions of remission, response, and symptom severity changes following antipsychotic treatment onset vary across studies, we tested model performance and generalizability across four different measures of outcome. Furthermore, we performed a thorough bias and benchmarking analysis of our models to determine whether model performances were influenced by ethnicity, medications, and baseline symptom severity. Finally, we thoroughly investigated the clinical utility of our models and critically evaluated their usefulness to psychiatrists and patients.

## Methods

### Sample

The established schizophrenia sample was taken from the Clinical Antipsychotic Trials of Intervention Effectiveness (CATIE): the protocol has already been detailed elsewhere and in the Supplementary Methods.^19^ The final sample consisted of 594 participants from 48 sites as a discovery sample and 83 participants from seven sites as an internal validation sample who had both three‐month and 12‐month outcome data (Fig. S1). 284 individuals with only a 3‐month follow‐up were kept back for the clinical scalability analysis. The First Episode Psychosis sample was taken from the European First Episode Schizophrenia Trial (EUFEST), the protocol of which has been described elsewhere.^20^ 323 participants had sufficient PANSS outcome data at 3 and 12 months (Fig. S2). No internal validation sample was created for this cohort due to the smaller sample size.

### Outcomes and features

We defined four prediction outcomes that are commonly used in the current literature at 3 months using the Positive and Negative Syndrome Scale (PANSS).^21^ A further analysis was conducted using the same outcomes at 12‐months. Total symptom severity was defined as the total PANSS score at 3 months. Percentage change in symptom severity from baseline was calculated by summing the 30 individual PANSS items at baseline and follow‐up, subtracting 30 from each total to rescale between 0 and 180,^22^ and then determined the percentage change. The binary 25% reduction in symptom severity was calculated by applying a cut‐off at 25% on the percentage change from baseline outcome.^23^ The 25% cut‐off was chosen because this is a commonly used definition of response in the literature as it equates to “minimal improvement” on the Clinical Global Impressions Scale^24^ while higher cut‐offs are often too stringent for individuals with established schizophrenia.^23^ This outcome can be subject to floor effects in participants who already have relatively low symptom severity, but in the two samples only 0.6% of participants in EUFEST and 1.5% of participants in CATIE had a PANSS score of 50 or below (the equivalent of “mild” symptoms across all domains) without seeing an increase in symptom severity at 3 months. We are therefore confident that this will not affect our results. RSWG remission was determined using the pre‐defined criteria without the 6‐month window.^25^

A full list of features can be found in Table S1. Predictive features consisted of 91 clinical, sociodemographic, and cognitive baseline variables that were present in both datasets. Single items and total scores from the Calgary Depression Scale for Schizophrenia^26^ and the Positive and Negative Syndrome Scale^27^ to capture both general symptom severity and specific clinical features that may independently predict treatment response. Other psychopathological measures included the Clinical Global Impression scale (CGI),^28^ psychiatric comorbidities harmonized from the Mini‐International Neuropsychiatric Interview (MINI)^29^ in EUFEST and the Structured Clinical Interview for DSM‐IV (SCID)^30^ in CATIE, and number of psychiatric hospitalizations. The antipsychotics olanzapine, quetiapine, and ziprasidone were common to both clinical trials and were therefore used as predictors, as well as antipsychotic dose: other antipsychotics were coded as “other”. The Rey Auditory Verbal Learning Test (RAVLT)^31^ and the Wechsler Adult Intelligence Scale (WAIS)^32^ digit symbol task were the only cognitive tests that were available in both datasets. Demographic and health variables included age, sex, ethnicity, unemployment, highest level of education of patients and their parents, height, weight, BMI, waist circumference, systolic blood pressure, diastolic blood pressure, and heart rate. All categorical variables were one‐hot encoded – for further details see Supplementary Methods.

### Machine learning analysis

The protocol was preregistered on the Open Science Framework (https://doi.org/10.17605/OSF.IO/DMYEH) with minor protocol deviations (Supplementary Methods). The study is compliant with TRIPOD+AI (Table S2). All machine learning analysis was performed in Neurominer 1.3.^33^ All models used a 10‐by‐10 repeated nested cross‐validation structure, 10 folds and 10 permutations in both the inner (CV1) and the outer (CV2) folds, to minimize the risk of model overfitting. Pre‐processing took place within each CV1 training partition to prevent data leakage. For all features, the data was first scaled between −1 and 1. Missing data was then imputed using k Nearest Neighbor imputation (where *k* = 7) using Euclidean distance. The data was then standardized to the mean of the sample. Due to the large number of sites and the discrepancies in samples sizes between sites (ranging from 3–41 participants), no site correction was performed. Classification models used an L2‐regularized L1‐loss linear Support Vector Machine algorithm.^51^ Regression models used an epsilon L2‐regularized, L1‐loss Support Vector Regression with a linear kernel. Model optimization is detailed in the Supplementary Methods. Model performance was measured in Balanced Accuracy (BAC), sensitivity, specificity and area under the curve (AUC) for classification models and Pearson's *r* for linear models.

Machine learning models were developed separately in the established schizophrenia and FEP datasets to predict each of the four outcome labels, and then externally validated in the other dataset. As an additional internal validation, the models developed in the established schizophrenia cohort were also validated in the 7 left‐out sites from the established schizophrenia study for comparison.

The significance threshold for all statistical analysis was *P* = 0.05. Model significance was determined by permuting the label 100 times to create a null distribution of model performances and then comparing the observed performance to this distribution. Feature importance was determined by sign‐based consistency: the number of times that the sign of the feature is consistent within an ensemble multiplied by the number of times that the variable was non‐zero.^34^ The cross‐validation ratio (the sum of the median feature weights across all CV1 folds divided by the standard deviation) was used in feature importance plots as a measure of the magnitude, direction and stability of the feature effect.

### Bias and benchmarking analysis

To investigate the performance of our models in subgroups of the discovery sample, we computed model performances separately in subgroups of the population with different sex at birth, ethnicity, antipsychotic medication, and changes in symptom severity (Supplementary Methods). For the ethnicity sensitivity analysis, the established schizophrenia sample was separated into White and Non‐White subgroups because there were too few participants in the Black, Asian and Other categories to use as separate subgroups. The first episode psychosis sample was not diverse enough to do this analysis (95% White) so we instead investigated any differences in model errors when validated in the established schizophrenia sample.

### Clinical utility analysis

For classification models that had a *P* > 0.05 in external validation, decision curve analysis was applied^35^ to estimate the net benefit of our model over a range of probability thresholds (the predicted probability at which a clinician would opt for the risk of treatment). Calibration curve analysis was used to evaluate how well the predicted probabilities of the positive class align with the observed outcomes.^36^ First, the predicted probabilities were partitioned into 10 static bins, corresponding to a 0.1 range of predicted probabilities. For each bin, we calculated the observed fraction of positive outcomes, which represents the proportion of instances where the true label was positive within that bin. Next, we plotted the calibration curve, which visualizes the relationship between the predicted probabilities (x‐axis) and the observed fraction of positives (y‐axis). Expected Calibration Error (ECE) measures the weighted average difference between the predicted probabilities and the observed outcome frequencies across all bins. An ECE of 0.1 or lower is generally considered well‐calibrated.^37^

To minimize the burden of data collection for both patients and clinicians, we reduced our models to only those variables that were found to be significant by sign‐based consistency in the discovery analysis. Total symptom scores were also removed from this analysis as they would require the whole clinical scale to be administered. These models were tested on 284 individuals from the established schizophrenia sample who were not included in the original analysis (Fig. S1). Finally, the classification and linear models were applied to predict the same outcome in the same sample at 12 months after baseline.

The study was approved by the institutional review board at each site for both CATIE and EUFEST, and written informed consent was obtained from the patients or their legal guardians.

## Results

### Model performance and generalizability

**Fig. 1:**
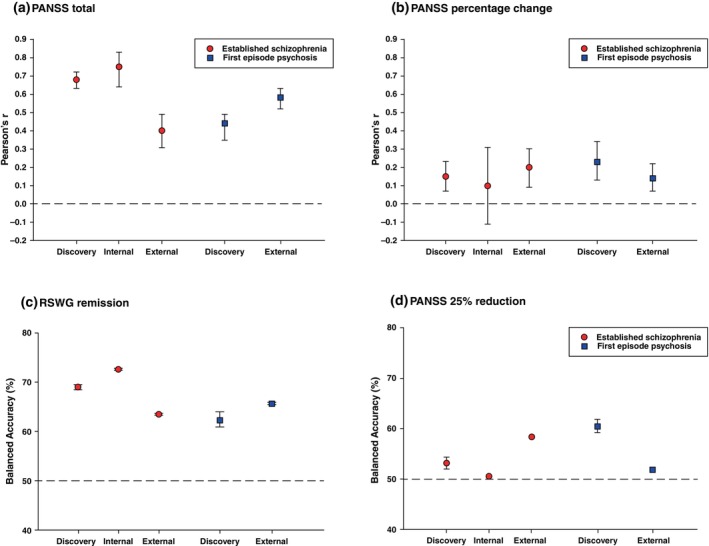
The performance of models predicting treatment outcome at 3 months across all definitions of response. (a) total symptom severity, (b) Percentage reduction in symptom from baseline, (c) Remission defined by the Remission in Schizophrenia Working Group (RSWG) Criteria, (d) Remission defined as a 25% reduction in total symptom severity. From left to right: the red graphs represent model performance in the established schizophrenia discovery sample, validation in the established schizophrenia internal validation sample and external validation in the FEP sample. The blue graphs represent the FEP discovery model, and its external validation in the established schizophrenia sample. Model performance of linear outcomes in (a) and (b) is measured by Pearson's *r* and for classification outcomes (c) and (d) performance is measured in Balanced Accuracy. The reference line marks a Pearson's *r* of 0 for linear outcomes and chance level performance at 50% for the classification outcomes. Error bars represent 95% confidence intervals: in some cases they are very narrow and may be smaller than the plotting symbols.

**Fig. 2:**
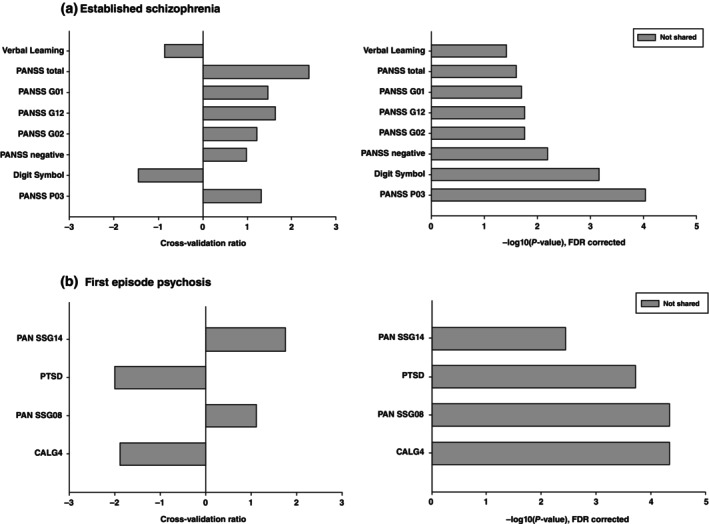
The significant predictors of the total symptom severity models for: (a) the established schizophrenia cohort, and (b) the FEP cohort. The left‐hand graphs represent the cross‐validation ratio of each feature. The right‐hand graphs show the −log10 of the FDR‐corrected *P*‐value. Darker bars represent variables that were common to both the established schizophrenia and first episode psychosis models, while the lighter bars represent features that were only in one model. Established schizophrenia: Verbal learning: Rey‐Auditory Verbal Learning Test, PANSS total: total Positive and Negative Syndrome (PANSS) scale score at baseline, PANSS G01: somatic concern, PANSS G12: Lack of judgment and insight, PANSS G02: Anxiety, PANSS negative: negative symptom score on the PANSS, Digit Symbol: the digit symbol task from the Wechsler adult intelligence scale, PANSS P03: Hallucinations. First episode psychosis: PANSS G14: Poor impulse control, PTSD: Post‐traumatic stress disorder, PANSS G08: Uncooperativeness, CALG4: Guilty ideas of reference on the Calgary Depression Scale for Schizophrenia.

Key demographics for all samples are presented in Table 1. Linear model discovery and validation performances are shown in Fig. 1 and Table S3. Total psychotic symptom severity was well‐predicted in the established schizophrenia cohort (*r* = 0.68, *P* < 0.001). This model was successfully validated in both an internal validation sample (*r* = 0.75, *P* < 0.001) and the external validation FEP sample (*r* = 0.4, *P* < 0.001). Top features were verbal learning score, total PANSS score, PANSS G01: somatic concern, PANSS G12: Lack of judgment and insight, PANSS G02: Anxiety, total PANSS negative score, the digit symbol task, and PANSS P03: Hallucinations (Fig. 2). The model developed in the FEP cohort had a lower discovery performance (*r* = 0.44, *P* < 0.001) but externally validated with a higher performance in the established schizophrenia sample (*r* = 0.58, *P* < 0.001). Top features of the model were PANSS G14: Poor impulse control, PTSD: Post‐traumatic stress disorder, PANSS G08: Uncooperativeness, and CALG4: Guilty ideas of reference on the Calgary Depression Scale for Schizophrenia (Fig. 2).

**Table 1:** Comparison of key variables in the established schizophrenia discovery and internal validation cohorts and the first episode psychosis cohort

	Established schizophrenia discovery	Established schizophrenia internal validation	*P*‐value	First episode psychosis	*P*‐value
Age (mean (SD))	41.3 (11.2)	41.9 (10.6)	0.68	26.0 (5.6)	<0.001
Sex (% male)	71.3%	88.0%	0.003	56.3%	<0.001
Baseline total symptom severity (mean (SD))	75.7 (17.8)	69.1 (15.8)	0.001	88.9 (20.0)	<0.001
PANSS total 3 months (mean (SD))	67.4 (17.5)	64.5 (17.2)	0.15	56.5 (16.0)	<0.001
Percent change in symptom severity 3 months (mean (SD))	13.6 (42.6)	9.4 (41.4)	0.39	53.4 (27.2)	<0.001
RSWG remission 3 months (% remission)	30.5%	30.1%	0.94	67.8%	<0.001
PANSS 25% reduction 3 months (% remission)	36.5%	31.3%	0.35	85.8%	<0.001
Ethnicity (% white)	64.6%	60.2%	0.43	95.7%	<0.001
Olanzapine (% taking)	27.0%	33.7%	0.20	24.8%	0.45
Quetiapine (% taking)	21.4%	16.8%	0.34	20.1%	0.66
Ziprasidone (% taking)	11.1%	8.5%	0.46	15.5%	0.06
Perphenazine (% taking)	17.5%	18.1%	0.89	‐	‐
Risperidone (% taking)	23.1%	22.9%	0.98	‐	‐
Haloperidol (% taking)	‐	‐	‐	19.1%	‐
Amisulpride (% taking)	‐	‐	‐	20.4%	‐
Dose (mean (SD))	107.3 (177.1)	86.7 (176.2)	0.35	103.5 (168.2)	0.38
Antidepressants	32.1%	25.0%	0.21	2.50%	2.1 × 10^−15^
Anxiolytics/hypnotics	29.4%	21.4%	0.14	43.7%	1.5 × 10^−5^
Anti‐Parkinson's drugs	20.9%	15.5%	0.27	11.8%	5.5 × 10^−4^
Comorbid depression	25.9%	28.9%	0.56	6.8%	2.2 × 10^−12^
Comorbid anxiety	9.7%	8.4%	0.7	10.5%	0.71
Comorbid PTSD	4.8%	6.0%	0.65	0.3%	2.1 × 10^−4^
Comorbid substance	31.0%	38.6%	0.16	22.2%	0.006

*P*‐values were derived from comparisons of the other two datasets with the established schizophrenia discovery sample. Significance threshold *P* < 0.05.

PANSS, Positive and Negative Syndrome Scale; RWSG, Remission in Schizophrenia Working Group.

The percentage change in symptom severity was poorly predicted in the established schizophrenia cohort (*r* = 0.15, *P* < 0.001) and externally validated in the FEP sample with a small increase in performance (*r* = 0.2, *P* < 0.001). Percentage change in symptom severity was also weakly predicted in the FEP cohort (*r* = 0.23 *P* < 0.001), with a lower but significant external validation performance (*r* = 0.14 *P* < 0.001).

**Fig. 3:**
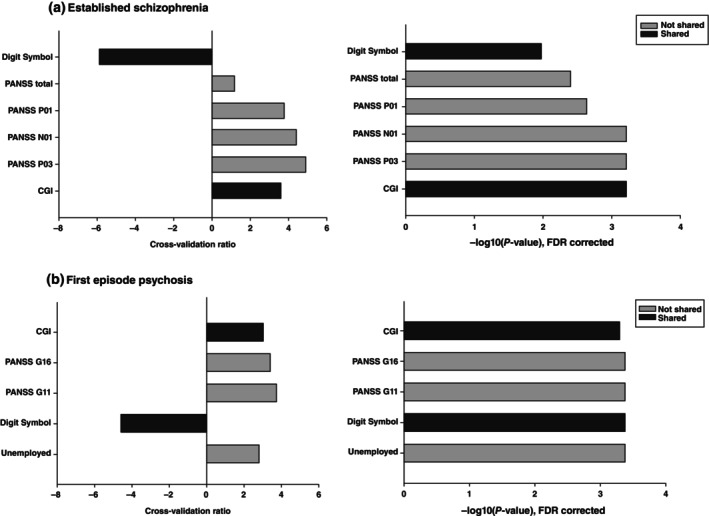
The significant predictors of the RWSG remission models for: (a) the established schizophrenia cohort, and (b) the First Episode Psychosis cohort. The left‐hand graphs represent the cross‐validation ratio of each feature. The right‐hand graphs show the −log10 of the FDR‐corrected *P*‐value. Darker bars represent variables that were common to both the established schizophrenia and first episode psychosis models, while the lighter bars represent features that were only in one model. Established schizophrenia: Digit Symbol: the digit symbol task from the Wechsler adult intelligence scale, PANSS total: total Positive and Negative Syndrome Scale (PANSS) score at baseline, PANSS P01: Delusions, PANSS N01: Blunted Affect, PANSS P03: Hallucinations, CGI: Clinician Global Impressions severity scale. First episode psychosis: PANSS P02: Conceptual disorganization, Unemployed: currently unemployed, Education – years: total years in education.

RSWG remission was predicted in the established schizophrenia sample with a Balanced Accuracy (BAC) of 69.0% (*P* < 0.001) and successfully validated in both the internal (BAC = 72.6%, *P* < 0.001) and external (BAC = 63.5%, *P* < 0.001) validation samples (Fig. 1, Table S4). Important features were the digit symbol task, total PANSS score, PANSS P1: Delusions, PANSS N01: Blunted Affect, PANSS P03: Hallucinations, and the Clinician Global Impressions severity scale (Fig. 3). RSWG remission was also predicted in the FEP sample with a BAC of 62.4% (*P* < 0.001) and externally validated in the established schizophrenia cohort with a similar BAC of 65.7%, (*P* < 0.001). Important features were the Clinician Global Impressions severity scale, PANSS G16: Active social avoidance, PANSS G11: poor attention, the digit symbol task, and unemployed at baseline (Fig. 3).

The 25% reduction in symptom severity was predicted weakly in both samples, but could not be externally validated (Fig. 1, Table S4).

### Investigating clinical utility

**Fig. 4:**
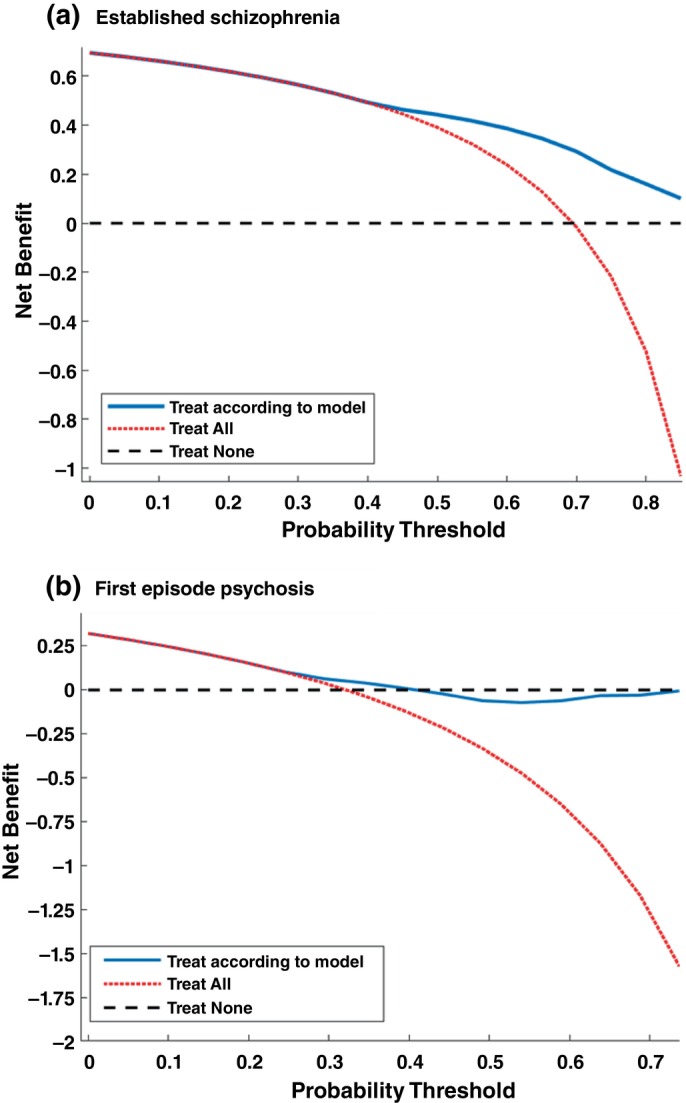
Decision curve analyses for the RSWG criteria in: (a) the established schizophrenia cohort, and (b) the First episode psychosis cohort.

**Fig. 5:**
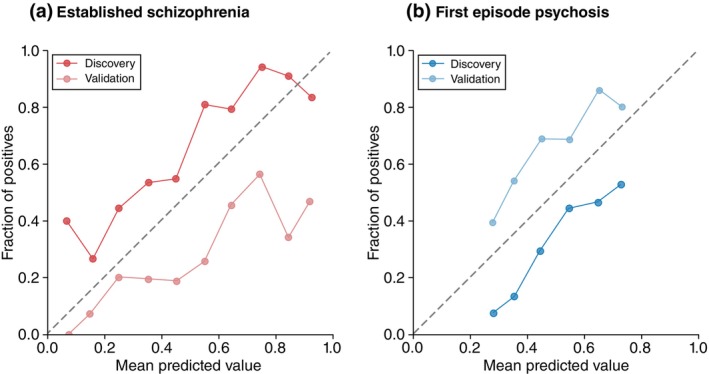
Calibration curves for: (a) the established schizophrenia cohort, and (b) the first episode psychosis cohort. The mean predicted value at each decile (x‐axis) is plotted against the actual frequency on non‐remission cases given the model's predicted probabilities.

Our models predicting RSWG remission in the established schizophrenia sample had a superior net benefit for probability thresholds between 0.5 and 0.9 (Fig. 4) in decision curve analyses compared to “treat all” and “treat none” conditions. The FEP model had significant net benefit only for probability thresholds between 0.3 and 0.4. The established schizophrenia model showed moderate calibration at the discovery level (ECE = 0.16), but poorer calibration at the validation level (ECE = 0.23). The majority of points in the calibration curve were above the diagonal for the discovery model, indicating that the models tend to underestimate the risk of non‐remission, and below the line for the validation model, indicating that the model over‐estimate the risk of non‐remission in the FEP sample (Fig. 5). The FEP model showed moderate calibration with an over‐estimation of non‐remission risk at the discovery level (ECE = 0.18), and moderate calibration with an under‐estimation of non‐remission risk at the validation level (ECE = 0.18).

We further investigated whether scaling our model that were only the single item features that were significant in either the established schizophrenia or the FEP model was feasible to reduce the time needed for data collection in 284 established schizophrenia participants who were not used in the discovery analysis (Fig. S1). The model predicting total symptom severity used Verbal learning, PANSS G01 (somatic concern), PANSS G12 (Lack of judgment and insight), PANSS G02 (Anxiety, the digit symbol score, and PANSS P03 (Hallucinations) form the established schizophrenia model, and PANSS G14 (Poor impulse control), PTSD, PANSS G08 (Uncooperativeness), and CALG4 (Guilty ideas of reference on the Calgary Depression Scale for Schizophrenia) from the FEP model (Fig. 2). The model performance was decreased compared to the full model but still highly significant (*r* = 0.53, *P* < 0.001). For the model predicting RSWG remission we used PANSS P01 (Delusions), PANSS N01 (Blunted Affect), and PANSS P03 (Hallucinations) from the established schizophrenia model, and PANSS G16 (Active social avoidance), PANSS G11 (poor attention), and Unemployed from the FEP model (Fig. 3). The model performance was also significant (BAC = 65.3%).

Finally, we investigated whether our models also predicted the total symptom severity and RSWG remission outcomes at 12‐months. The distribution of outcomes at 12 months is shown in Table S5. The models developed in the established schizophrenia sample predicted these outcomes with a slightly lower performance (*r* = 0.53 and BAC = 66.3% respectively, Table S6). However, the models developed in the FEP sample were much less effective at predicting 12‐month outcomes (*r* = 0.26, BAC = 57.1%, Table S6).

### Bias and benchmarking analysis

Our binary RSWG model developed in the established schizophrenia cohort performed significantly better in males (BAC = 71.0%) than females (BAC = 63.3%, *P* = 3 × 10^−6^) (Table 2, Fig. S3). The RSWG model developed in the FEP sample followed a similar pattern (female BAC = 61.8%, male BAC = 66.1%, *P* = 3.33 × 10^−4^, Table 2, Fig. S4). To see whether these differences might be caused by higher response rates in one sex, we investigate whether the rates of the model's false non‐remission predictions differed between sexes (Table S7). We found no difference in the percentage of false non‐remission predictions in the established schizophrenia model (*n* = 198, *P* = 0.23) and a non‐significant trend in the FEP model (*n* = 114, females 73.5% *vs*. males 56.9%, *P* = 0.068). The total symptom severity model developed in the established schizophrenia cohort also performed significantly better in males (male r_z_ = 0.64, female r_z_0.40, *P* = 1.03 × 10^−19^, Fig. S3), while the FEP model showed no differences (Fig. S4).

**Table 2:** Performance of models developed in the established schizophrenia and FEP cohorts in subgroups

		Male	Female	*P*‐value
Established schizophrenia	Subgroup sample size	434 (73.1%)	160 (26.9%)	N/A
	Total symptom severity	0.40	0.64	1.03 × 10^−19^
	RWSG remission (% remission)	71.0%	63.3%	3.0 × 10^−6^
First episode psychosis	Subgroup sample size	182 (56.3%)	141 (43.7%)	N/A
	Total symptom severity	0.28	0.32	0.05
	RWSG remission (% remission)	66.1%	61.8%	3.33 × 10^−4^

The median model performance across the 100 outer‐fold partitions reported in Balanced Accuracy for the RSWG model and z‐adjusted Pearson's *r* for total symptom severity to account for the effect of subgroups. *P*‐values were derived using a Mann–Whitney test on the model performances across the 100 outer folds.

The binary RSWG remission model developed in the established schizophrenia cohort had a small but non‐significant difference in performance between White (BAC = 69.5%) and Non‐White (BAC = 66.7%) subgroups (*P* = 0.06), while the total symptom severity model in the same cohort saw small but significant differences (*P* = 0.007, Table 3, Fig. S5). When validating the FEP models in the established schizophrenia cohort we saw small but significant differences between subgroups (Table S8).

**Table 3:** Performance of models developed in the established schizophrenia and FEP cohorts in ethnicity subgroups

		White	Non‐White	*P*‐value
Established schizophrenia	Subgroup sample size	384 (64.6%)	210 (35.4%)	N/A
	Total symptom severity (r_z_)	0.40	0.37	0.007
	RWSG remission (BAC)	69.5%	66.7%	0.06

The median model performance across the 100 outer‐fold partitions reported in Balanced Accuracy for the RSWG model and z‐adjusted Pearson's *r* for total symptom severity to account for the effect of subgroups. *P*‐values were derived using a Mann–Whitney test on the model performances across the 100 outer folds. This analysis was not possible for the FEP sample because of the lack of diversity so the model's validation in the established schizophrenia sample was compared instead (see Table S8).

The RSWG remission model developed in the established schizophrenia cohort had significant differences between the performance in participants on risperidone (BAC = 74.4%) and perphenazine (BAC = 63.1%, *P* = 0002) but not between any other medications (Table 4, Fig. S6). The total symptom severity model had higher performances in ziprasidone (r_z_ = 0.36) and olanzapine (r_z_ = 0.36) compared to the other medications. The RSWG model developed in the FEP sample saw significant subgroup differences between quetiapine (BAC = 53.5%) and haloperidol (BAC = 75.0%, *P* = 0.002), quetiapine (BAC = 53.5%) and olanzapine (BAC = 70.0%, *P* = 0.02), and between haloperidol (BAC = 75.0%) and ziprasidone (BAC = 61.2%, 0.0006) (Table 5, Fig. S7). The total symptom severity model performed better in participants taking olanzapine (r_z_ = 0.27) and amisulpride (r_z_ = 0.23) compared to participants taking other medications. We compared the number of false non‐remission cases across the five medications for the binary RSWG models to see if any of the medications were more efficacious and therefore led to a better‐than‐predicted outcome. However, there were no significant differences between the medications (Tables S9 and S10).

**Table 4:** Performance of models developed in the established schizophrenia cohort in subgroups randomized to different antipsychotic medications

	Ziprasidone	Olanzapine	Quetiapine	Risperidone	Perphenazine	*P*‐value
Subgroup sample size	66 (11.1%)	160 (27.0%)	127 (21.4%)	137 (23.1%)	104 (17.5%)	N/A
Total symptom severity (r_z_)	0.36	0.36	0.27	0.27	0.26	**8.5 × 10** ^**−14**^
RWSG remission (BAC)	74.1%	68.1%	67.7%	74.4%	63.1%	**0.01**

The median model performance across the 100 outer‐fold partitions reported in Balanced Accuracy for the RSWG model and *z*‐adjusted Pearson's *r* for total symptom severity to account for the effect of subgroups. *P*‐values were derived using a Kruskal–Wallis test on the model performances across the 100 outer folds. Pairwise comparisons are shown in Fig. S6.

**Table 5:** Performance of models developed in the first episode psychosis cohort in subgroups randomized to different antipsychotic medications

	Ziprasidone	Olanzapine	Quetiapine	Haloperidol	Amisulpride	*P*‐value
Subgroup sample size	50 (15.5%)	80 (24.8%)	65 (20.1%)	62 (19.1%)	66 (20.4%)	N/A
Total symptom severity (r_z_)	0.13	0.27	0.13	0.12	0.23	2.8 × 10^−15^
RWSG remission (BAC)	61.2%	70.0%	53.5%	75.0%	66.7%	0.0001

The median model performance across the 100 outer‐fold partitions reported in Balanced Accuracy for the RSWG model and z‐adjusted Pearson's *r* for total symptom severity to account for the effect of subgroups. *P*‐values were derived using a Kruskal–Wallis test on the model performances across the 100 outer folds. Pairwise comparisons are shown in Fig. S7.

We also investigated whether our models were only predictive to the autocorrelation of baseline and 3‐month symptom severity, by comparing performances across patients who had a 20% increase or decrease in symptoms compared to those who did not. The RSWG remission model developed in the established schizophrenia cohort had a small but significant difference in performance in individuals who did not change (BAC = 74.0%) and those who saw a 20% reduction in symptom severity (BAC = 70.8%, *P* = 0.01) (Table 6, Fig. S8). The model had a lower but still significant performance in individuals who saw a 20% increase in symptom severity (BAC = 66.7%). The total symptom severity model performed much better in individuals who did not have a significant change (r_z_ = 0.65) compared to those who saw a 20% reduction (r_z_ = 0.42). The model had a poor performance in those who had a 20% increase in symptom severity (r_z_ = 0.13). The RSWG model developed in the FEP cohort had similar performances in the group that saw no change (BAC = 66.7%) and the group that saw a 20% reduction (BAC = 65.5%), but a much lower performance in the group who has an increase in symptom severity (BAC = 50%, *P* = 0.01) (Table 6, Fig. S9). The total symptom severity model performed best in individuals who saw a 20% reduction (r_z_ = 0.39), followed by those with no change (r_z_ = 0.31) and then those who saw a 20% increase (r_z_ = 0.23). We also compared model performance across different subgroups defined by quartiles of baseline symptom severity (Table S11, Figs. S10 and S11). In the total symptom severity model trained on the established schizophrenia cohort, model performance was significantly higher in quartiles 1 and 4 (*r* = 0.52–0.59) compared with quartiles 2 and 3 (*r* = 0.16–0.23). The FEP total symptom severity model showed a similar pattern. Conversely, the model predicting RSWG remission in the established schizophrenia sample had better performances in quartiles 2 and 3 (BAC = 60 = 62.3%) compared with quartiles 1 and 4 (BAC = 50%–57.1%). The FEP RSWG model performed best in quartiles 1 and 3 (BAC = 66.7%–68.7%) and less well in quartiles 2 and 4 (BAC = 53.6%–58.3%).

**Table 6:** Performance of the RSWG and total symptom severity models across subgroups defined by their change in symptom severity from baseline to 3 months

		>20% decrease	No change	20% increase	*P*‐value
Established schizophrenia	Subgroup sample size	65 (10.9%)	261 (43.9%)	268 (45.1%)	N/A
	Total symptom severity (r_z_)	0.13	0.65	0.42	3.6 × 10^−51^
	RWSG remission (BAC)	66.7%	74.0%	70.8%	0.0001
First episode psychosis	Subgroup sample size	5 (1.5%)	32 (9.9%)	286 (88.5%)	N/A
	Total symptom severity (r_z_)	50%	66.7%	65.5%	0.01
	RWSG remission (BAC)	0.23	0.31	0.39	3.0 × 10^−11^

A comparison of model performances in subgroups of individuals who saw a 20% reduction in symptom severity from baseline to 3‐month follow‐up, people who saw a 20% increase in symptoms, and those who saw a less than 20% change. The median model performance across the 100 outer‐fold partitions reported in Balanced Accuracy for the RSWG model and z‐adjusted Pearson's *r* for total symptom severity to account for the effect of subgroups. *P*‐values were derived using a Kruskal–Wallis test on the model performances across the 100 outer folds. Pairwise comparisons are shown in Figs. S8 and S9.

## Discussion

We have developed machine learning models to forecast changes in disease severity at 3 months following commencement of antipsychotic medication. Models predicting total symptom severity and Remission in Schizophrenia Working Group (RSWG) remission successfully generalized across geographically and disease‐stage‐wise distinct patient populations. Our models predicting RSWG remission were reasonably well calibrated, showed superior net benefit for some thresholds of risk and could be reduced to a parsimonious, clinically scalable set of eight to nine variables. A comprehensive bias and benchmarking analysis demonstrated that sex, ethnicity, and medication affected model performance.

Generalizable prediction models for antipsychotic response are required for implementing personalized care for psychosis patients. However, although several studies have previously used clinical and sociodemographic data to predict disease severity changes in psychosis,^38^, ^39^, ^40^, ^41^, ^42^, ^43^, ^44^, ^45^ only two of these studies successfully validated their models in external data,^44^, ^45^ while other evidence suggests that these models do not generalize well to other samples.^17^ Here we present the first models of antipsychotic response to generalize across continents and disease stages, representing a crucial step forward for precision psychiatry. Current research often focuses exclusively on the first episode psychosis (FEP) group with very little focus on later psychosis stages, whereas our models performed well in both earlier and later stages of schizophrenia, which is vital for individuals whose psychosis is detected at a later stage. However, it is important to note that our models performed very differently depending on the definition of treatment outcome: models predicting total symptom severity and RSWG remission had moderate‐to‐good performances, but percentage change in symptom severity was much less well predicted. The current literature on antipsychotic response uses a wide range of outcome definitions including criteria based on PANSS symptomatology,^42^, ^44^ functioning scales such as the Clinical Global Impression (CGI)^43^ and the Global Assessment of Functioning (GAF),^38^ and other outcomes such as treatment discontinuation^41^ or clozapine use.^45^ Our results demonstrate the importance of outcome selection for precision psychiatry, and the need for response definitions that are predictable, generalizable across different psychosis populations, and patient relevant going forward.

Many of our top features were related to symptom severity, which has been previously associated with antipsychotic response.^46^ Our sensitivity analyses suggest that this is partially due to autocorrelation of symptom severity between baseline and 3 months, especially in the total symptom severity models, because model performances were significantly higher in individuals who did not have significant symptom change between the two timepoints. However, the RSWG model had good performances across different subgroups symptom severity change, suggesting that autocorrelation does not completely explain our predictive findings. This is further supported by our analysis stratifying individuals based on baseline symptom severity: if our performances were solely driven by baseline symptom severity predictive performance would be strongest at the extremes (low baseline scores who would already be near the remission threshold or very high baseline scores who would be very unlikely to remit). Instead, superior performance in the central quartiles indicates the model leverages additional predictive information to distinguish remitters from non‐remitters among patients with similar baseline severity.

Interestingly some individual symptoms were selected by the model. Some, such as delusions and negative symptoms, may reflect overall illness severity, while others, such as lack of insight and uncooperativeness, have been previously linked to non‐compliance.^47^ Our cognitive variables were also highly predictive: while previous research does not directly implicate cognition in response to antipsychotics, a recent paper found associations between verbal memory and digit symbol tasks and antipsychotic response that were significant before multiple testing correction.^48^ This association may be because poor cognition is also often associated with worse psychopathology Unemployment was also associated with poorer treatment response, which is consistent with prior research showing that social and occupational dysfunction is both a consequence and predictor of more severe or treatment‐resistant illness.^49^, ^50^ Although PTSD was present in our FEP model, only one person had a PTSD diagnosis in this sample, and this is therefore likely to be statistical noise. Our reduced models with eight to nine single items showed slightly reduced but promising performances, showing the potential to further scale and optimize feature selection for ease of use. For example, the PANSS is not currently used by clinicians and would require training and 30–60 min per patient,^51^ whereas it may be possible to measure single symptoms without administering the whole interview.

The generalizability of our models across two populations does not necessarily mean that they perform equally well for all patients. Meehan and colleagues emphasize in their systematic review that nearly all current clinical prediction models in psychiatry have a high likelihood of bias.^15^ We have therefore performed, to our knowledge, the most comprehensive bias and benchmarking analysis of any prediction model in psychosis, and our results emphasize the importance of looking at the effects of factors such as sex, ethnicity, medication and symptom severity. Even though sex at birth was included as a predictor, our models generally performed better in males than females. The established schizophrenia sample was 71% male so these differences may reflect the lack of female representation in the sample. There was a larger rate of false non‐remissions predictions in women for the FEP model, although this did not quite reach statistical significance: the differences in model performance may therefore also be due to increased response rates in women, which is consistent with current literature,^52^ leading to an unexpected better outcome. Men and women also often experience differences in diagnosis in treatment in schizophrenia, so there may be a systemic bias in our data.^53^ Similarly, our ethnicity subgroup analysis saw a small but significant increase in model performance in White populations compared to non‐White populations for the linear total symptom severity model, but only trend effects for the RSWG model. This must be further investigated in more diverse samples where it is possible to perform a more fine‐grained analysis of model performances across a range of different ethnicities and ancestries. Ethnicity sensitivity analysis is especially important because clinical trial data often lacks diversity, which can exacerbate existing disparities in healthcare when respectively trained clinical prediction models are introduced into clinical care.^54^ There is a lot of racial bias in diagnosis of schizophrenia with a higher rate of misdiagnosis in non‐White populations.^55^

Different antipsychotics also had differing effects on model performance, despite this data being included among the predictors. Prediction models for antipsychotic treatment response are often developed on clinical trials where participants are randomized to different antipsychotics,^38^, ^41^ so it is important to understand these differences in performance as they may indicate that the model might perform in a clinical setting depending on which antipsychotic the patient was prescribed. We did not find any significant differences between the number of false non‐remission and false remission predictions, so this is unlikely to be because some medications may perform better than the model expects. We also did not see lower performances in medication subgroups that were not coded as predictors: risperidone and haloperidol had high model performances. Further work is therefore required to understand the complex effects of medication on clinical prediction models.

### Clinical implications

Predicting which individuals with psychosis will have the greatest risk of non‐response to first‐line antipsychotic treatments has exciting clinical potential. Current clinical guidelines for clozapine, the antipsychotic most commonly used for treatment‐resistant schizophrenia, recommend treatment initiation only after two failed trials of other antipsychotics due to I's aggressive side effect profile and need for regular monitoring.^56^ However, although evidence suggests that earlier treatment initiation of clozapine is associated with better functioning and increased remission of negative symptoms,^57^, ^58^ clozapine treatment is significantly delayed, often for years.^59^ Our model could therefore be used to identify non‐responders who could be prescribed clozapine treatment at an earlier stage. Our promising 12‐month outcomes predictions in the established schizophrenia sample further increase its utility for determining chronic non‐responders who would most benefit from earlier clozapine intervention. Although our models had moderate performances ranging from 62.4%–69% Balanced Accuracy, a paper by Jin and colleagues suggests that prediction models for treatment resistance in psychosis with accuracies of over 60% would have meaningful economic impact.^60^ Furthermore, our decision curve analyses demonstrated that the performances of the models developed in the established schizophrenia cohort may still be sufficient in individuals with a greater than 50% risk of non‐remission likelihood.^60^ In other fields of medicine probability thresholds of 50% are rare as they imply that the intervention is highly undesirable relative to missing a true non‐responder. However, given the high burden of clozapine treatment^61^ it is possible that clinicians would require a high level of certainty that an individual would be a non‐responder. The model is therefore currently most applicable for identifying a small group of patients who are the greatest risk of non‐response for treatment escalation, particularly those who have already failed one or more antipsychotic trials. Our discovery RSWG remission models were moderately well‐calibrated with ECE values between 0.16 and 0.18, which are comparable to other prediction models in the field.^62^

However, several aspects of our results suggest the models are not currently ready for clinical translation. The range of probability tthresholds for which our models had significant net benefit was very narrow for the first episode psychosis models, suggesting that these models may not currently have sufficiently high performance for clinical translation. Furthermore, our model showed systematic calibration differences with an underestimation of non‐remission risk in established schizophrenia and an overestimation of non‐remission risk in first episode psychosis. This pattern is consistent with clinical expectations, as FEP patients generally show higher rates of remission with antipsychotics, whereas long‐term patients more frequently present with treatment resistance. Therefore, local recalibration or group‐specific risk adjustment may be necessary prior to clinical deployment. The differences in model performance in sex, ethnicity, and medication subgroups must be studied further in the more diverse datasets. Furthermore, our results demonstrate that both the performance and generalizability of our models are heavily influenced by the definition of treatment outcome. Given the current diversity in outcomes in the current literature, a consensus is required on which outcomes are both patient‐relevant and generalizable. The involvement of experience experts in this consensus process is key for increasing the adoption likelihood of the respective models in clinical care.^63^

### Strengths and limitations

A key strength of our study was the rigorous methodology. The use of nested cross‐validation and the minimal use of hyperparameter optimization significantly reduced model overfitting and allowed us to objectively assess the generalizability of our models. Our bias and benchmarking analyses are, to our knowledge, the most rigorous of all current prediction models in psychiatry. One of the main limitations of our research is that antipsychotic treatment was not kept consistent across the study period. In the CATIE trial, participants who showed non‐response or cumbersome side effects were moved to another medication, while in EUFEST the antipsychotic remained the same but supplementary treatment with a second antipsychotic was allowed. This meant that it was not possible to develop individual prediction models for different antipsychotics individually. Furthermore, some participants were also on other psychotropic medications, but this could not be accounted for in our models due to the complex prescription changes throughout the trials. An additional limitation is the lack of ethnic diversity in our datasets, particularly the European EUFEST cohort. We were also limited in our choice of variables: since we aligned both samples to the same feature set there is the potential that some predictive information was lost in factors such as duration of untreated psychosis, the number of previous antipsychotic trials, and premorbid adjustment.^46^ We also were not able to correct for site or report any site‐based metrics due to the large number of sites, some of which had very few participants. Finally, we only considered measures of response that are centered around total illness severity. Future work could explore whether predictive patterns differ for positive *versus* negative symptoms, as suggested by Lee and colleagues^64^ since antipsychotics are much less effective in negative symptoms and can even in some cases worsen these symptoms.^65^

## Conclusions

In conclusion, we demonstrate a robust framework for rigorously training and benchmarking models for precision psychiatry. Our models predicting antipsychotic response were found to be generalizable across patient populations with profound differences in disease stage and geographical location. However, increased diversity in psychosis datasets is vital to ensure our models are fair and equitable. Furthermore, an international consensus on which outcome definitions should be used for predictive modeling and how their real‐life ascertainment is implemented within an urgently needed paradigm shift toward a measurement‐based care approach in psychiatry is needed.

## Funding

No funding source had any role in the study design, data collection, data analysis, data interpretation, writing, or submission of this report. The CATIE clinical trial was funded by the National Institutes of Mental Health grant N01 MH090001‐06 (JL). EUFEST is funded by the European Foundation for Research in Schizophrenia.

## Disclosure statement

Authors declare that they have no competing interests.

## Supporting information


**Data S1.** Supporting Information.

## Data Availability

The initial protocol for this study is pre‐registered on the Open Science Framework Registries (https://doi.org/10.17605/OSF.IO/DMYEH). CATIE clinical trial can be accessed *via* the NIMH Data Archives. The EUFEST dataset is available from Professor Rene Kahn upon request.
