# Supplementary material for: Ensuring generalizability and clinical utility in mental health care applications: Robust artificial intelligence‐based treatment predictions in diverse psychosis populations
Source: Psychiatry Clin Neurosci. 2025 Nov 6;80(1):64–75. doi: 10.1111/pcn.13914 (PMC12757767; doi:10.1111/pcn.13914)
Supplement: Supplementary file 1 — Data S1. Supporting Information. [file PCN-80-64-s001.docx]

**Supplementary Methods**

**Study Design**

*Established schizophrenia sample*

The established schizophrenia sample is taken from the Clinical Antipsychotic Trials of Intervention Effectiveness (CATIE) (*1*). Briefly 1460 individuals with schizophrenia were recruited from 57 U.S clinical sites between October 2001 and December 2004. The study was approved by the institutional review board at each site, and written informed consent was obtained from the patients or their legal guardians. Patients were included if they were aged 18-65, currently or previously met Diagnostic and Statistical Manual of Mental Disorders (DSM-IV) criteria for schizophrenia, have a condition appropriate for oral medication treatment, and have capacity to consent. Exclusion criteria were a diagnosis of schizoaffective disorder, mental retardation, pervasive developmental disorder, delirium, dementia, amnesia, or other cognitive disorders, documented serious adverse reactions or non-response to any of the study medications, first episode psychosis, current or past clozapine treatment, requiring long acting injectables, pregnancy and breastfeeding, contraindications for any of the study drugs, serious medical conditions, cardiac conditions, interacting medications, and taking any investigational drug within 30 days of the baseline assessment. A wide spectrum of patients with schizophrenia enrolled in the study, ranging from partially remitted outpatients to exacerbated inpatients. Participants were initially randomised to oral olanzapine, perphenazine, quetiapine, risperidone, or ziprasidone under double-blind conditions in Phase 1 and were followed up for 18 months. Patients with tardive dyskinesia (n=231) were not offered perphenazine but were randomised to one of the other four study medications (Phase 1A). Patients who discontinued a drug in phase 1/1A then progressed to Phase 2, where the study doctor allocated them to the “efficacy pathway” or the “tolerability pathway” depending on their reason for discontinuation. The efficacy pathway (2E) compared the effects of open-label clozapine to double-blind olanzapine, quetiapine, or risperidone. The tolerability pathway (2T) compared double-blinded treatment with olanzapine, quetiapine, risperidone, or ziprasidone. Patients who discontinued their medication at Phase 2 then entered Phase 3, where a study medication was recommended based on clinician and patient choice. Baseline data was obtained prior to the onset of treatment, and participants attended monthly visits for 18 months. At 3 months 85.7% were in Phase 1/1A, 12.1% were in Phase 2 and 1.9% in Phase 3. At 12 months, 61.5% of the remaining sample were in Phase 1/1A, 21.7% were in Phase 2, and 16.8% were in Phase 3. At 18 months, 58.3% were in Phase 1/1A, 19.3% were in Phase 2, and 22.4% were in Phase 3. The overall drop-out rate was 50.1%. 680 participants attended a 3-month and 12-month follow-up, and 284 had only a 3-month follow-up. 3 participants were removed for having more than 20% missing data, leaving 677 participants with both follow-ups for the main analysis.

*First episode psychosis sample*

The first episode psychosis sample was taken from the European First Episode Schizophrenia Trial (EUFEST) (*2*). 498 participants with first episode schizophrenia, schizoaffective, or schizophreniform disorder were recruited between December 2002 and January 2006 from 50 sites across Europe and Israel. The trial complied with the Declaration of Helsinki and was approved by local ethics committees: written informed consent was given by all participants. Participants who met inclusion criteria were 18-40 years of age, and met DSM-IV criteria for schizophrenia, schizoaffective, or schizophreniform disorder. Exclusion criteria were positive symptoms for more than 2 years, antipsychotic treatment for more than 2 weeks in one year or 6 weeks lifetime and known intolerance on contraindication to study drugs. Participants were randomised to oral haloperidol, amisulpride, olanzapine, quetiapine, or ziprasidone. Adjunctive treatment with additional antipsychotics was allowed within specified dose ranges. 91% had a diagnosis of schizophrenia, and 9% a diagnosis of schizoaffective disorder. Baseline data was obtained prior to treatment initiation, and participants attended visits at Data was collected at 0·5, 1, 1·5, 2, 3, 6, 9, and 12 months after baseline. 408 participants had data at 3 months and 343 participants had data at 12 months. The dropout rate was 31.2%.

**Feature processing**

The number of correct answers in each of the first three trials of the RAVLT as well as the total number of correct answers across the three trials were used in the model. Ethnicity in CATIE was defined as White, Black, Asian, Native American, Pacific or Hispanic, while in EUFEST the categories were White, Black, Asian and other: to harmonise, the Native American, Hispanic and Pacific categories in CATIE were combined into one variable “Other”. To determine other psychiatric diagnoses, the Mini-International Neuropsychiatric Interview (MINI) (*3*) was used in EUFEST and the Structured Clinical Interview for DSM-IV (SCID) (*4*) was used in CATIE. Common variables were selected from these two questionnaires, although it should be noted that diagnoses on the SCID are from the last 5 years, while the MINI measures lifetime occurrence of the disorder. The common diagnoses were depression, alcohol dependence, alcohol abuse, drug dependence, drug abuse, OCD, agoraphobia, panic disorder, PTSD, social phobia, and specific phobia. Two additional variables were calculated to determine the number of comorbid mood disorders (the total number of diagnoses of depression, OCD, agoraphobia, panic disorder, PTSD, social phobia or specific phobia) and substance use disorders (the total number of alcohol dependence/abuse and drug dependence/abuse disorders). The highest degree of education for patients and their parents were recorded in both samples. In CATIE, highest degree of education was coded as follows: “Advanced degree completed [e.g. Ph.D.]”, “Advanced degree courses, not graduated, college graduate”, “college graduate and some Master's level”, “Community college or technical school degree”, “Did not complete high school”, “GED/High school diploma”, “Master's degree completed” and “Some college, did not graduate”. In EUFEST, highest degree of education was coded as follows: “university (finished)”, “university (unfinished)”, “professional training (finished)”, “professional training (unfinished)”, “highschool (finished)”, “highschool (unfinished)”, and “less than highschool”. Four harmonised variables were extracted from this information: “Bachelors (finished)”: (“Advanced degree completed [e.g. Ph.D.]”, “college graduate and some Master's level”, “Master's degree completed” from CATIE, and “university (finished)” from EUFEST), “Bachelors (unfinished)”: (“Some college, did not graduate” from CATIE, and “university (unfinished)” from EUFEST), “highschool (finished)”: (“Community college or technical school degree”, “GED/High school diploma” from CATIE, and “highschool (finished)” from EUFEST), and “highschool (unfinished)” (“Did not complete high school” from CATIE, and “highschool (unfinished)” and “less than highschool” from EUFEST.

**Machine learning analysis**

Hyperparameter optimisation occurred in the CV1 folds to strictly separate the process of model training from the process of model application conducted in the outer CV partitions. 11 Slack parameters were trialled during hyperparameter optimisation: 0,015625, 0.03125, 0.0625, 0.125, 0.25, 0.5, 1, 2, 4, 8, 16. The tolerance parameter was set at 0.01 and weighting of the hyperplane for uneven group sizes was used by inverse ratio because Support Vector Machines are suboptimal with unbalanced groups. Six values of the epsilon parameter were tests: 0.05, 0.1 0.15, 0.2, 0.25, 0.3. The tolerance was set to 0.01. For feature selection, a wrapper with greedy forward feature selection was applied to the CV1 test and training data with 80% early stopping and 10% feature stepping. Significant features of the model were reported based on sign-based consistency *(52)*. 95% confidence intervals were derived based on the CV2 performances. Site correction was not used, because both samples had a large number of sites, some of which had very few participants: it was therefore decided that site correction might introduce unwanted heterogeneity.

**Benchmarking analysis**

Subgroups for analysis were determined as follows. For the ethnicity sensitivity analysis, the established schizophrenia sample was separated into White and Non-White subgroups because there were too few participants in the Black, Asian and Other categories to use as separate subgroups. The first episode psychosis sample was not diverse enough to do this analysis (95% White) so we instead investigated any differences in model performance when validating the model in the established schizophrenia model. We compared the predicted scores and the difference between the observed and predicted scores for the linear models and the decision scores and number of misclassifications for the linear models. In the medication sensitivity analysis, model performances were compared in subgroups who were randomised to different antipsychotics. The percentage of false remitters and false non-remitters for each medication was compared using a Chi-squared test. To determine whether our model was only effective in people who did not see any symptom severity change from baseline, we compared model performances in people who had a greater than 20% increase in symptom severity from baseline to 3 months, those who had a greater than 20% decrease in symptom severity and those who saw less than 20% change. Model performances were also compared across different quartiles of baseline symptom severity.

Model performance in subgroups of the discovery samples was ascertained by extracting the model performances (Balanced Accuracy for binary models and Pearson’s r for linear models) for each CV2 fold separately for each subgroup. Pearson’s r values are strongly affected by sample size so each performance value was z-transformed to account for this. We then compute the median performance for each subgroup, and the distribution of the performances in the different subgroups were compared using a Mann-Whitney test for the ethnicity analysis, and Kruskall-Wallis tests for the medication and symptom severity analysis.

For linear models we compared the distribution of predicted scores and the difference between observed and predicted symptom severity from the external validation between subgroups using a Mann-Whitney test. For the binary models we compared the model decision scores from the external validation between subgroups using a Mann-Whitney test, and the model misclassifications using a Chi-squared test.

**Protocol deviations**

The initial protocol for this study is pre-registered on the Open Science Framework Registries (https://doi.org/10.17605/OSF.IO/DMYEH). The focus of this paper was hypothesis 3 on the 3-month outcomes. At the time of registration, we did not have access to the EUFEST dataset, so the external validation does not feature in the pre-registration. After the paper by Chekroud et al (*5*) we added two additional outcomes (percentage change in PANSS and 25% reduction in PANSS) to more directly compare with their paper.

**Supplementary Figures**


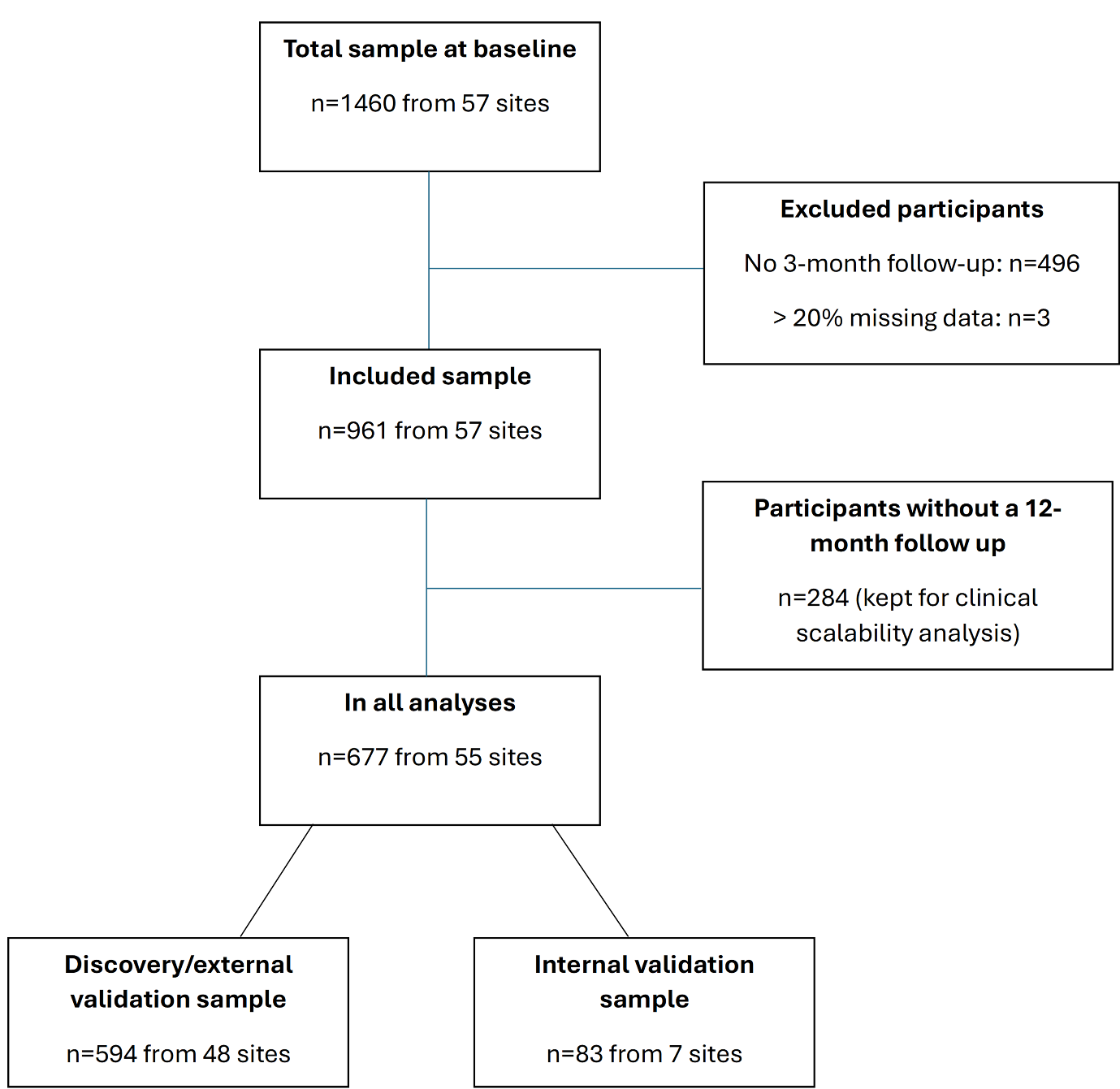


**Fig.S1. CONSORT chart showing the participant selection from the CATIE clinical trial.**


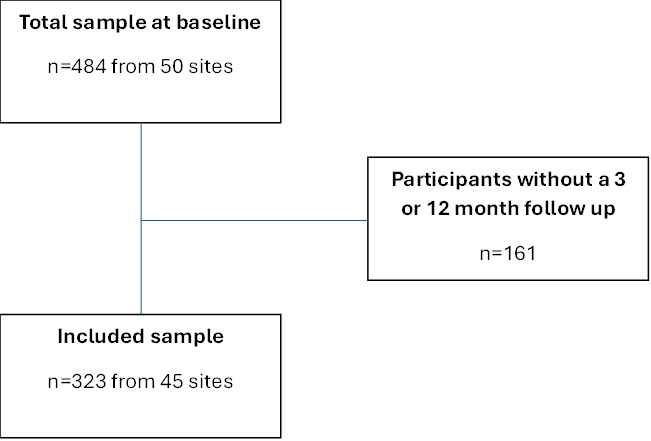


**Fig.S2. CONSORT chart showing the participant selection from the EUFEST clinical trial**

**
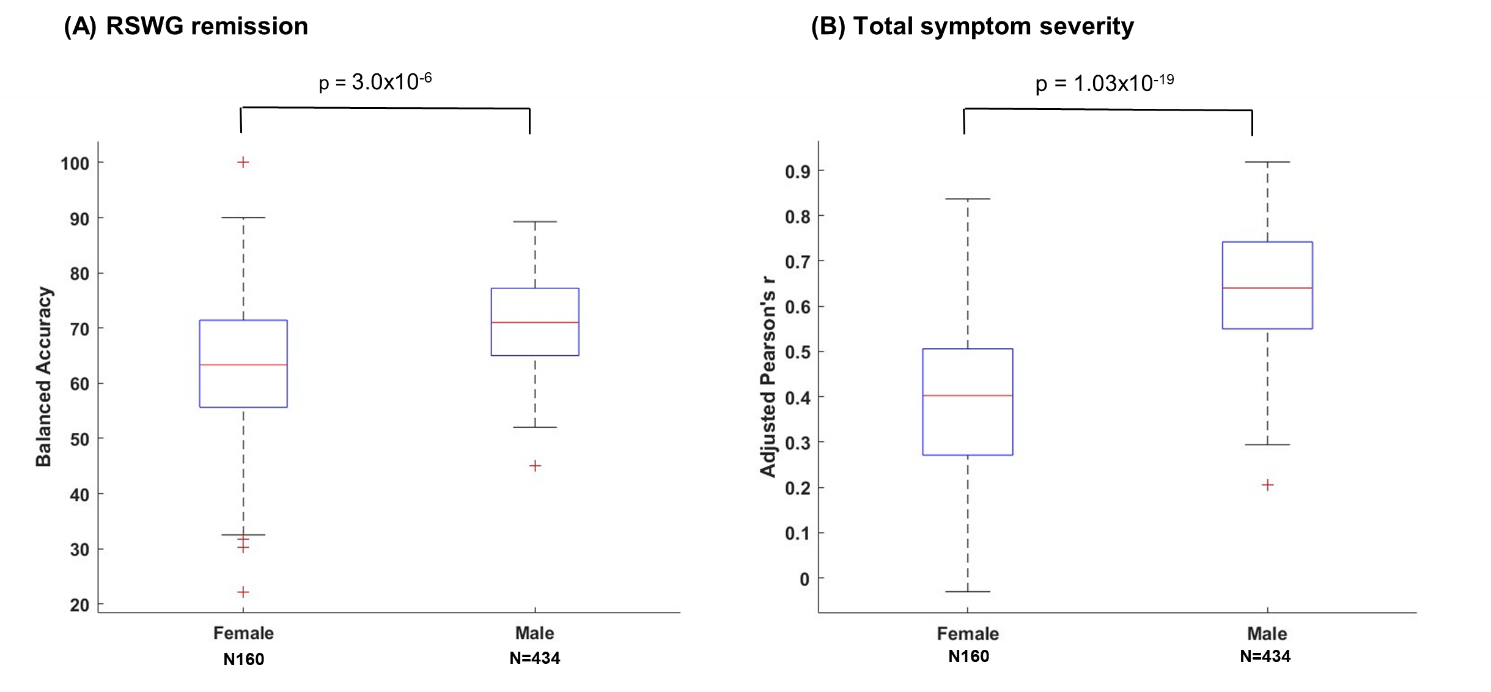
**

**Fig.S3 Performances of the models developed in the established schizophrenia sample in male and female sub-groups**

The median model performance across the 100 outer-fold partitions reported in Balanced Accuracy for the RSWG model and z-adjusted Pearson’s r for total symptom severity to account for the effect of subgroups. P-values were derived using a Mann-Whitney test on the model performances across the 100 outer folds.


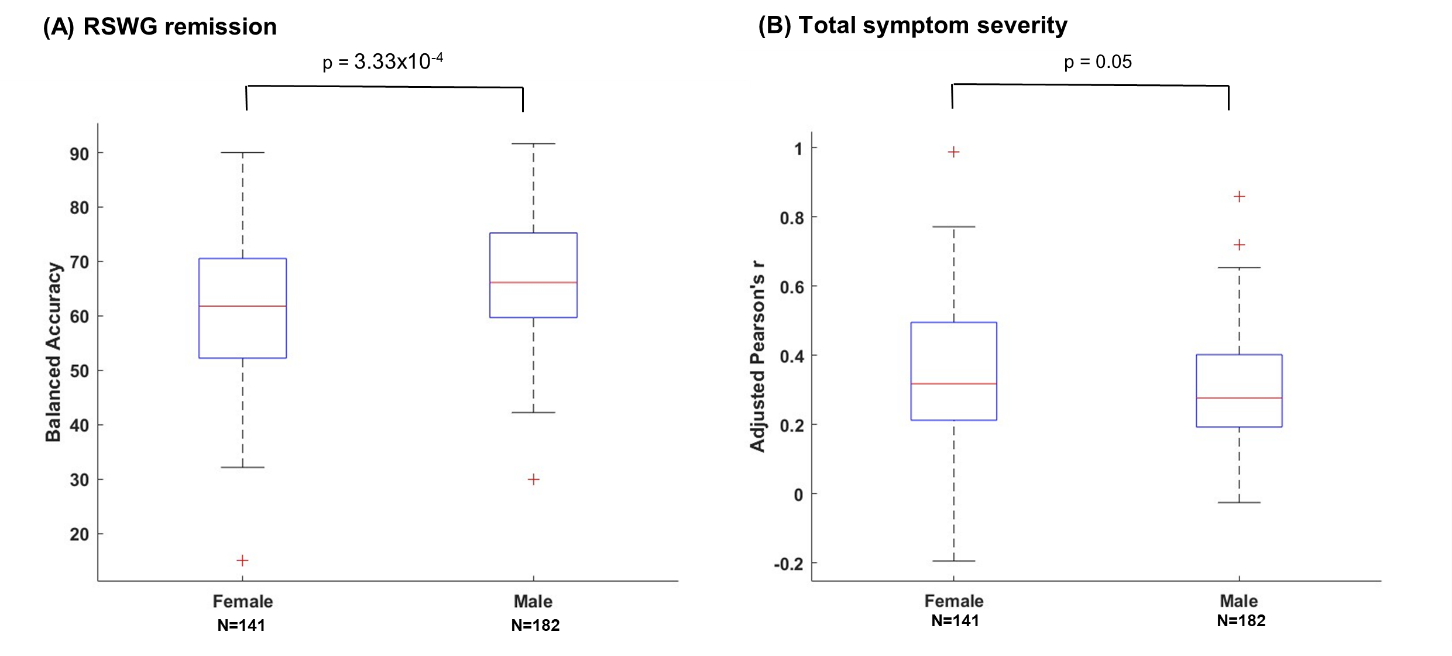


**Fig.S4 Performances of the models developed in the first episode psychosis sample in male and female sub-groups**

The median model performance across the 100 outer-fold partitions reported in Balanced Accuracy for the RSWG model and z-adjusted Pearson’s r for total symptom severity to account for the effect of subgroups. P-values were derived using a Mann-Whitney test on the model performances across the 100 outer folds

**
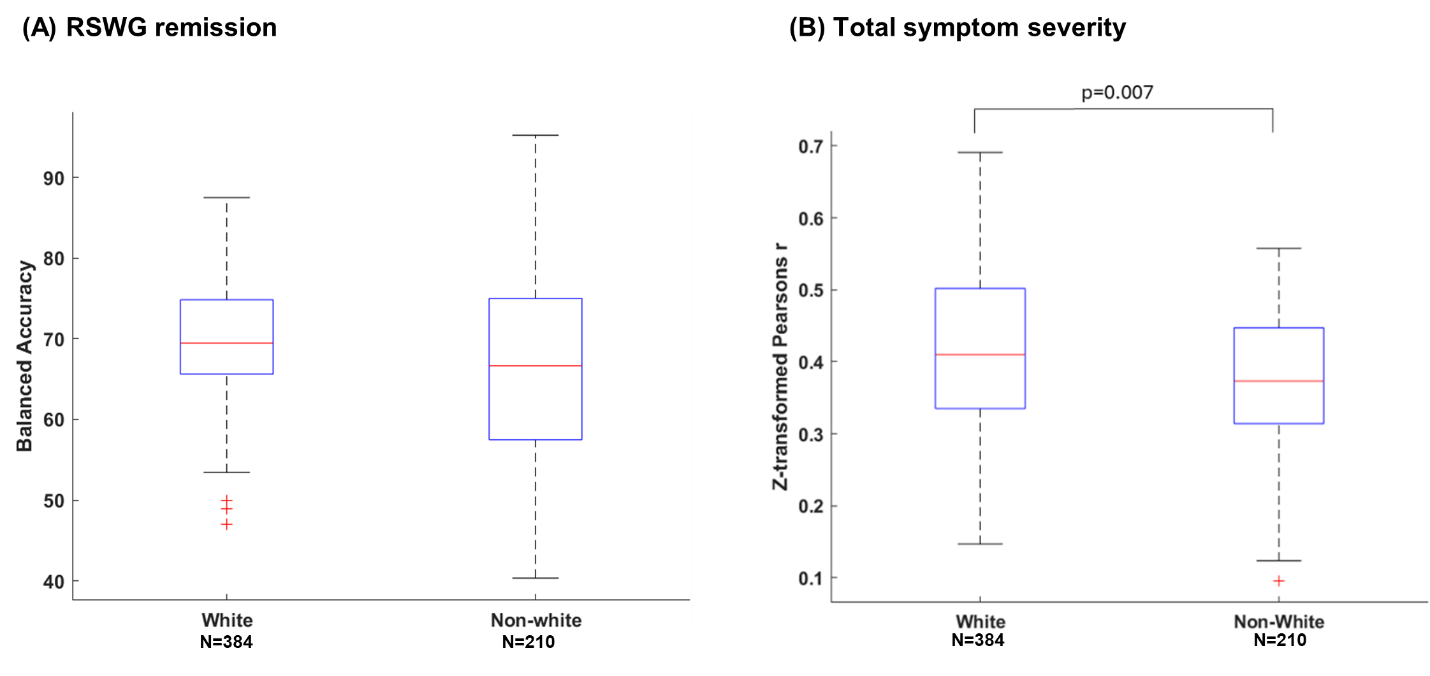
**

**Fig.S5 Performances of the models developed in the established schizophrenia White and non-White sub-groups**

The median model performance across the 100 outer-fold partitions reported in Balanced Accuracy for the RSWG model and z-adjusted Pearson’s r for total symptom severity to account for the effect of subgroups. P-values were derived using a Mann-Whitney test on the model performances across the 100 outer folds.


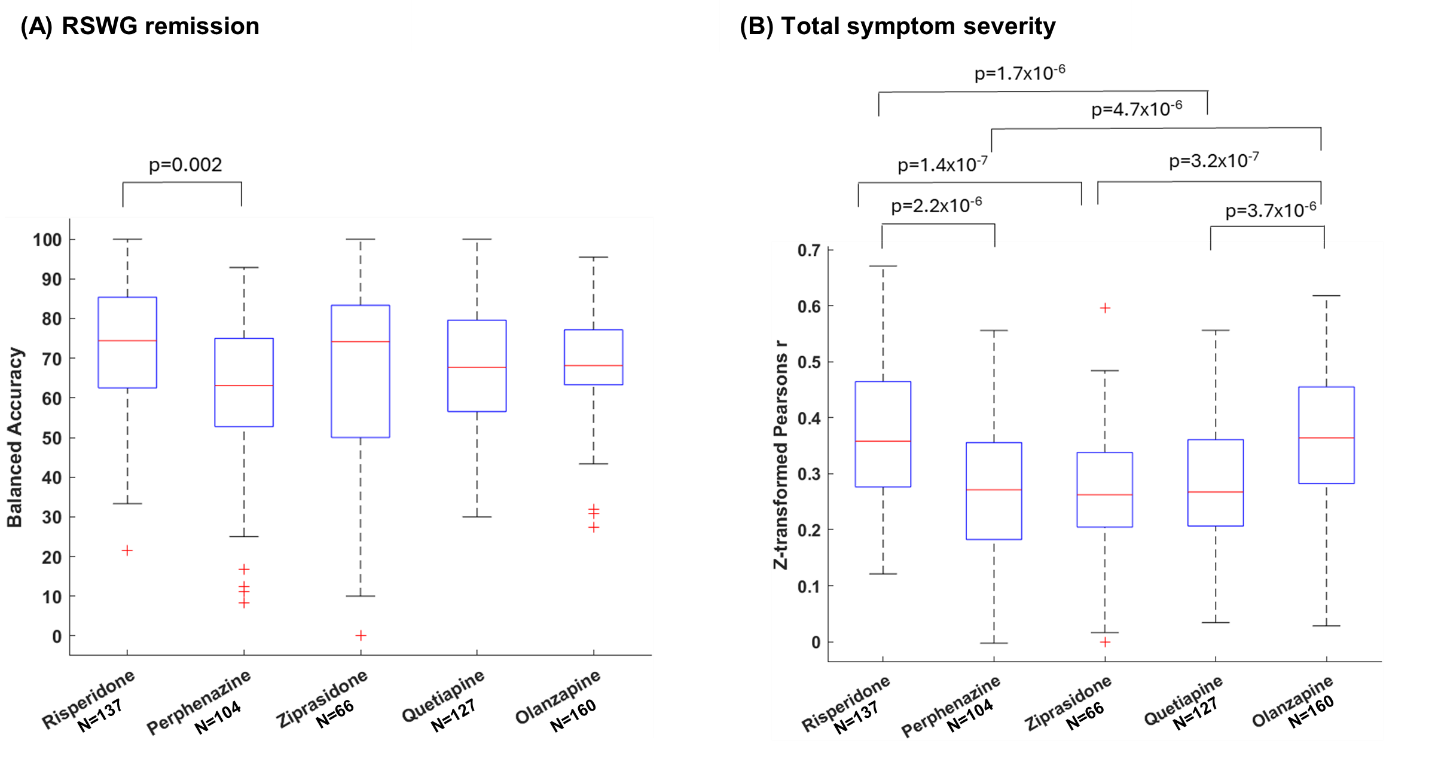


**Fig.S6 Performances of the models predicting total symptom severity and RSWG remission in the established schizophrenia sample in different medication groups.**

The median model performance across the 100 outer-fold partitions reported in Balanced Accuracy for the RSWG model and z-adjusted Pearson’s r for total symptom severity to account for the effect of subgroups. P-values were derived using a Mann-Whitney test on the model performances across the 100 outer folds.


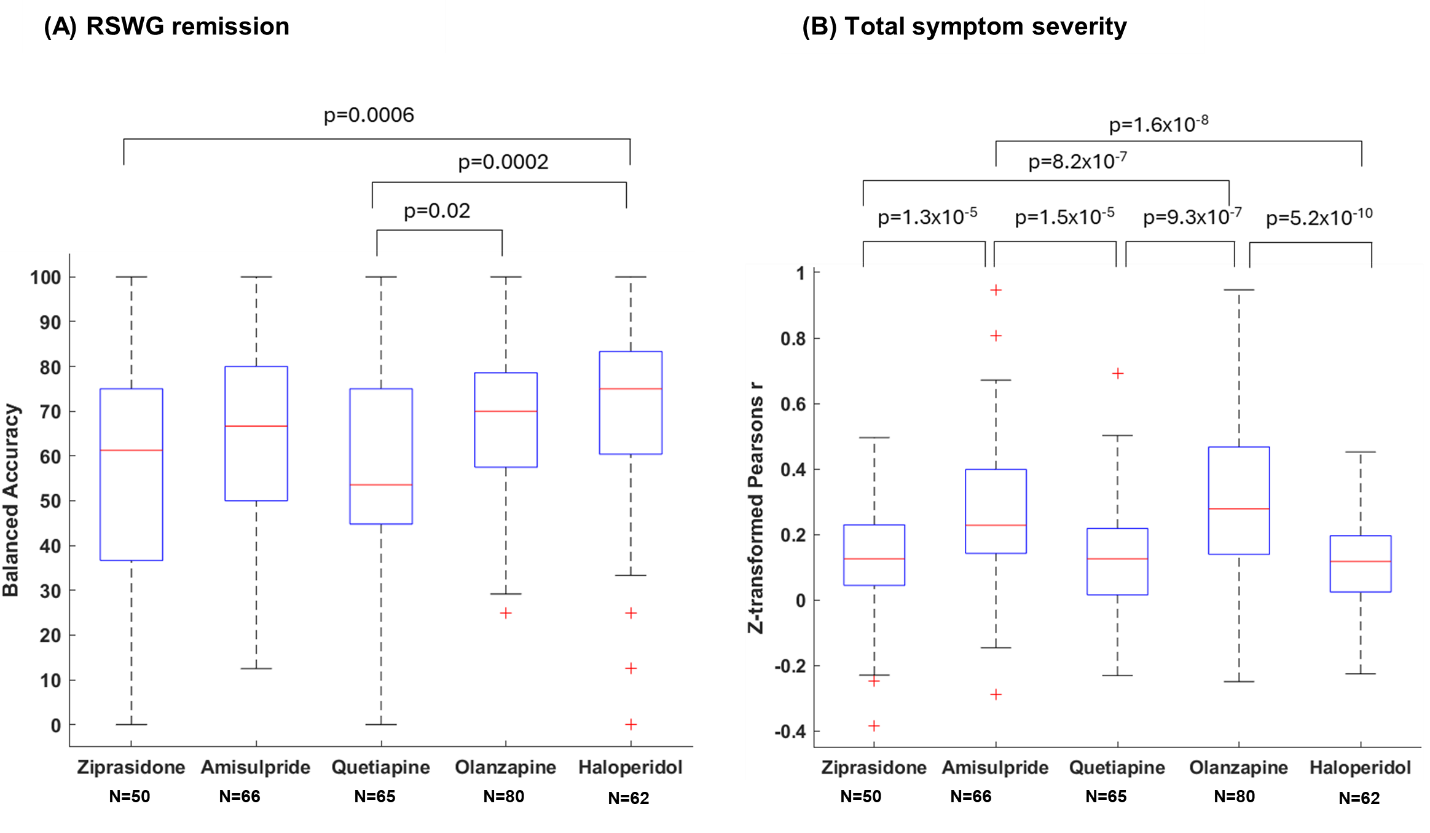


**Fig.S7 Performances of the models predicting total symptom severity and RSWG remission in the FEP sample in different medication groups.**

The median model performance across the 100 outer-fold partitions reported in Balanced Accuracy for the RSWG model and z-adjusted Pearson’s r for total symptom severity to account for the effect of subgroups. P-values were derived using a Mann-Whitney test on the model performances across the 100 outer folds.


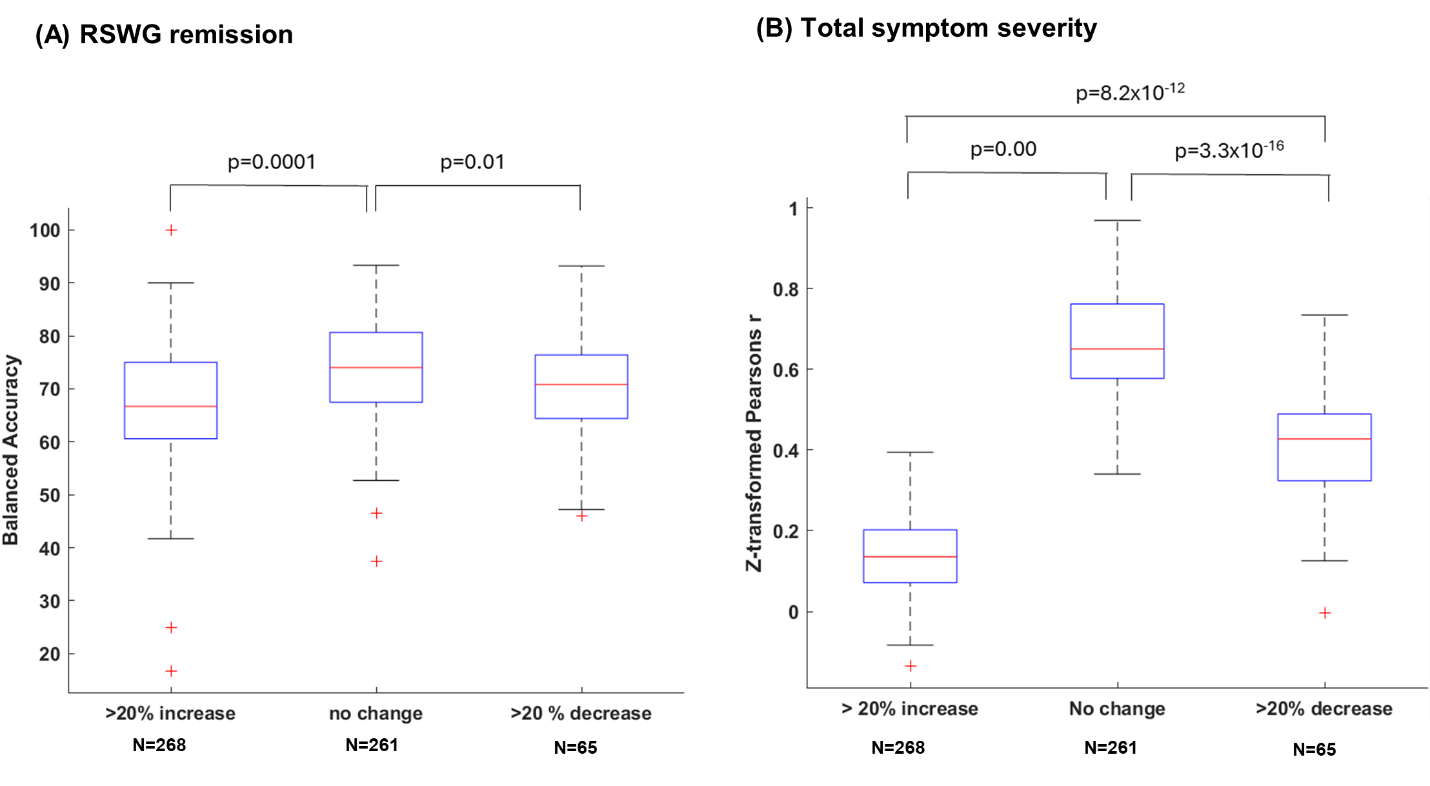


**Fig.S8 Performance of the RSWG and total symptom severity models in the established schizophrenia cohort across subgroups defined by their change in symptom severity from baseline to 3 months.**

A comparison of model performances in subgroups of individuals who saw a 20% reduction in symptom severity from baseline to three-month follow-up, people who saw a 20% increase in symptoms, and those who saw a less than 20% change.

The median model performance across the 100 outer-fold partitions reported in Balanced Accuracy for the RSWG model and z-adjusted Pearson’s r for total symptom severity to account for the effect of subgroups. P-values were derived using a Kruskall-Wallis test on the model performances across the 100 outer folds.


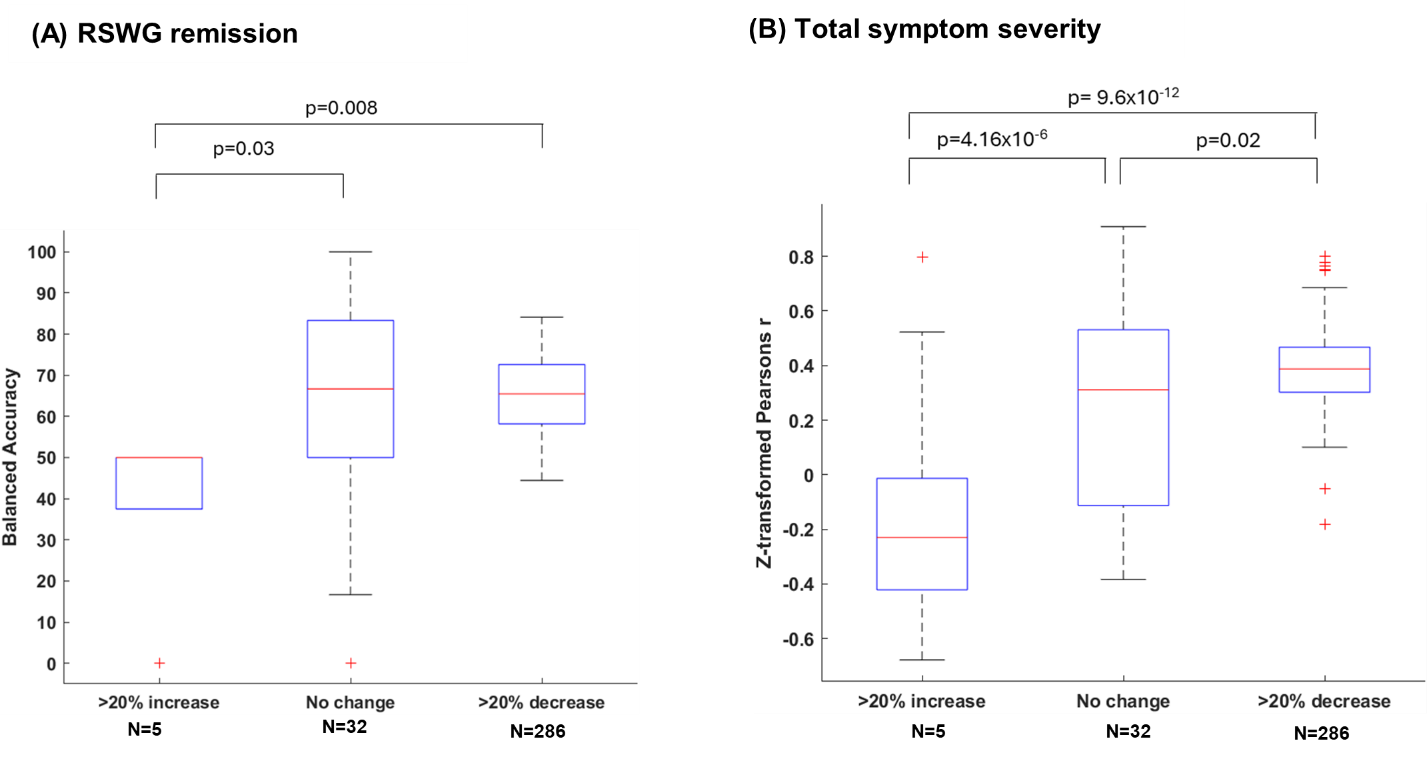


**Fig.S9 Performance of the RSWG and total symptom severity models in the first episode psychosis cohort across subgroups defined by their change in symptom severity from baseline to 3 months.**

A comparison of model performances in subgroups of individuals who saw a 20% reduction in symptom severity from baseline to three-month follow-up, people who saw a 20% increase in symptoms, and those who saw a less than 20% change.

The median model performance across the 100 outer-fold partitions reported in Balanced Accuracy for the RSWG model and z-adjusted Pearson’s r for total symptom severity to account for the effect of subgroups. P-values were derived using a Kruskall-Wallis test on the model performances across the 100 outer folds


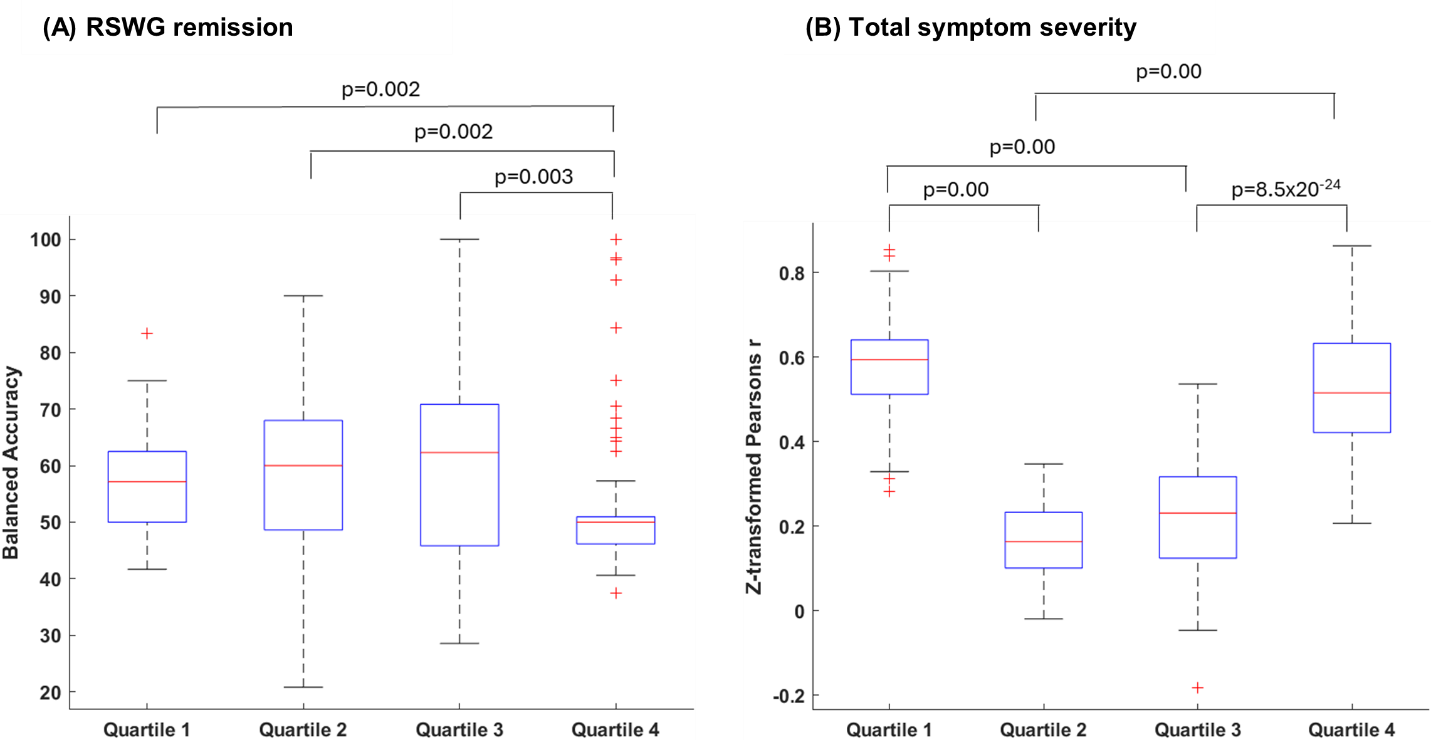


**Fig.S10 Performance of the RSWG and total symptom severity models in the established schizophrenia cohort across the four quartiles of symptom severity.**

The median model performance across the 100 outer-fold partitions reported in Balanced Accuracy for the RSWG model and z-adjusted Pearson’s r for total symptom severity to account for the effect of subgroups. P-values were derived using a Kruskall-Wallis test on the model performances across the 100 outer folds.


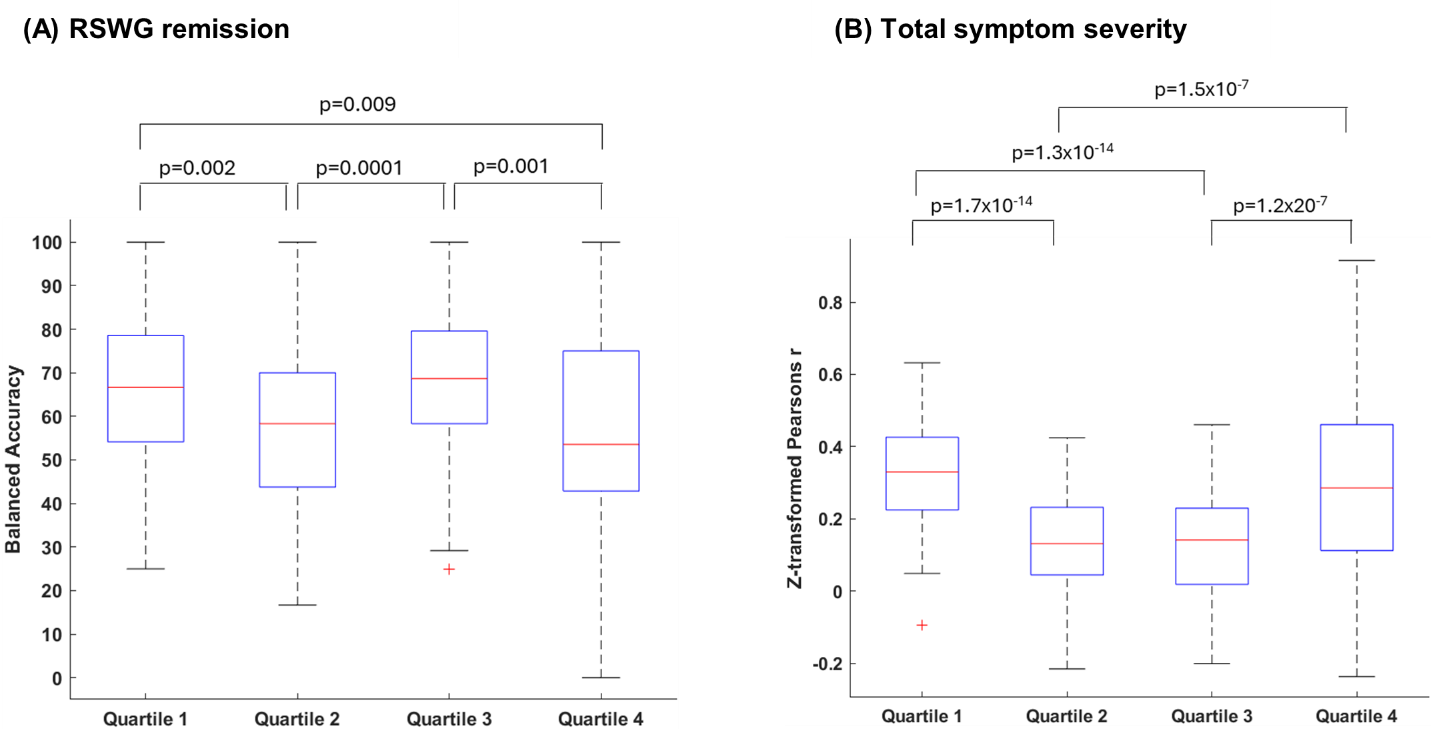


**Fig.S11 Performance of the RSWG and total symptom severity models in the first episode psychosis cohort across the four quartiles of symptom severity.**

The median model performance across the 100 outer-fold partitions reported in Balanced Accuracy for the RSWG model and z-adjusted Pearson’s r for total symptom severity to account for the effect of subgroups. P-values were derived using a Kruskall-Wallis test on the model performances across the 100 outer folds.

**Supplementary Tables**

| Variable | Description |
| --- | --- |
| Psychopathology | |
| CALG total | Total score on the Calgary Depression Scale for Schizophrenia |
| CALG1 | Calgary Depression Scale for Schizophrenia: Depression |
| CALG2 | Calgary Depression Scale for Schizophrenia: Hopelessness |
| CALG3 | Calgary Depression Scale for Schizophrenia: Self-depreciation |
| CALG4 | Calgary Depression Scale for Schizophrenia: Guilty ideas of reference |
| CALG5 | Calgary Depression Scale for Schizophrenia: Pathological guilt |
| CALG6 | Calgary Depression Scale for Schizophrenia: Morning depression |
| CALG7 | Calgary Depression Scale for Schizophrenia: Early wakening |
| CALG8 | Calgary Depression Scale for Schizophrenia: Suicide |
| CALG9 | Calgary Depression Scale for Schizophrenia: Observed depression |
| CGI | Clinician rated severity on the Clinical Global Impressions Scale |
| Hospitalisations | Number of hospitalisations in the last year |
| PANSS general | Positive and Negative Syndrome Scale: total general symptom score |
| PANSS negative | Positive and Negative Syndrome Scale: total negative symptom score |
| PANSS positive | Positive and Negative Syndrome Scale: total positive symptom score |
| PANSS total | Positive and Negative Syndrome Scale: total score |
| PANSSG01 | Positive and Negative Syndrome Scale: Somatic concern |
| PANSSG02 | Positive and Negative Syndrome Scale: Anxiety |
| PANSSG03 | Positive and Negative Syndrome Scale: Guilt feelings |
| PANSSG04 | Positive and Negative Syndrome Scale: Tension |
| PANSSG05 | Positive and Negative Syndrome Scale: Mannerisms and posturing |
| PANSSG06 | Positive and Negative Syndrome Scale: Depressions |
| PANSSG07 | Positive and Negative Syndrome Scale: Motor retardation |
| PANSSG08 | Positive and Negative Syndrome Scale: Uncooperativeness |
| PANSSG09 | Positive and Negative Syndrome Scale: Unusual thought content |
| PANSSG10 | Positive and Negative Syndrome Scale: Disorientation |
| PANSSG11 | Positive and Negative Syndrome Scale: Poor attention |
| PANSSG12 | Positive and Negative Syndrome Scale: Lack of judgement and insight |
| PANSSG13 | Positive and Negative Syndrome Scale: Disturbing of volition |
| PANSSG14 | Positive and Negative Syndrome Scale: Poor impulse control |
| PANSSG15 | Positive and Negative Syndrome Scale: Preoccupation |
| PANSSG16 | Positive and Negative Syndrome Scale: Active social avoidance |
| PANSSN01 | Positive and Negative Syndrome Scale: Blunted affect |
| PANSSN02 | Positive and Negative Syndrome Scale: Emotional withdrawal |
| PANSSN03 | Positive and Negative Syndrome Scale: Poor rapport |
| PANSSN04 | Positive and Negative Syndrome Scale: Passive/apathetic withdrawal |
| PANSSN05 | Positive and Negative Syndrome Scale: Difficulty in abstract thinking |
| PANSSN06 | Positive and Negative Syndrome Scale: Lack of spontaneity and flow of conversation |
| PANSSN07 | Positive and Negative Syndrome Scale: Stereotyped thinking |
| PANSSP01 | Positive and Negative Syndrome Scale: Delusions |
| PANSSP02 | Positive and Negative Syndrome Scale: Conceptual disorganisation |
| PANSSP03 | Positive and Negative Syndrome Scale: Hallucinations |
| PANSSP04 | Positive and Negative Syndrome Scale: Excitement |
| PANSSP05 | Positive and Negative Syndrome Scale: Grandiosity |
| PANSSP06 | Positive and Negative Syndrome Scale: Suspiciousness/persecution |
| PANSSP07 | Positive and Negative Syndrome Scale: Hostility |
| Medication | |
| Antipsychotic: Dose | Antipsychotic dose (mg) |
| Antipsychotic: olanzapine | Current olanzapine treatment |
| Antipsychotic: quetiapine | Current quetiapine treatment |
| Antipsychotic: ziprasidone | Current ziprasidone treatment |
| Comorbidities | |
| Agoraphobia | Diagnosis of agoraphobia |
| Alcohol abuse | Diagnosis of alcohol abuse |
| Alcohol dependence | Diagnosis of alcohol dependence |
| Comorbid mood disorder | Number of comorbid mood disorders |
| Comorbid substance disorder | Number of comorbid substance use disorders |
| Depression | Diagnosis of depression |
| Drug abuse | Diagnosis of drug abuse |
| Drug dependence | Diagnosis of drug dependence |
| OCD | Diagnosis of Obsessive-Compulsive Disorder |
| Panic disorder | Diagnosis of panic disorder |
| PTSD | Diagnosis of Post-Traumatic Stress Disorder |
| Social phobia | Diagnosis of social phobia |
| Specific phobia | Diagnosis of specific phobia |
| Sociodemographics | |
| Age | Participant age (years) |
| Ethnicity: Asian | Self-reported Asian ethnicity |
| Ethnicity: Black | Black self-reported ethnicity |
| Ethnicity: other | Self-reported other ethnicity |
| Ethnicity: White | Self-reported white ethnicity |
| Sex | Sex a birth |
| Education and employment | |
| Education: Highschool (finished) | Secondary education – completed |
| Education: Highschool (unfinished) | Secondary education – not completed |
| Education: Bachelors (finished) | University/college degree – completed |
| Education: Bachelors (unfinished) | University/college degree – not completed |
| Education: years | Total years in education |
| Parent education: high-school (finished) | Parent with secondary education – completed |
| Parent education: high-school (unfinished) | Parent with secondary education – not completed |
| Parent education: university (finished) | Parent with a university/college degree – completed |
| Parent education: university (unfinished) | Parent with a university/college degree – not completed |
| Unemployed | Currently unemployed |
| Physical health | |
| BMI | Body Mass Index |
| BP: Diastolic | Diastolic blood pressure |
| BP: Systolic | Systolic blood pressure |
| Heart rate | Heart rate |
| Height | Height (cm) |
| Waist | Waist circumference (cm) |
| Weight | Weight (kg) |
| Cognition | |
| Digit symbol | Total score on the digit symbol test from the Wechsler Adult Intelligence Scale |
| Verbal learning total | Number of correct responses across three trials of the Rey Auditory Verbal Learning Test |
| Verbal learning trial 1 | Number of correct responses on the first trial of the Rey Auditory Verbal Learning Test |
| Verbal learning trial 2 | Number of correct responses on the second trial of the Rey Auditory Verbal Learning Test |
| Verbal learning trial 3 | Number of correct responses on the third trial of the Rey Auditory Verbal Learning Test |

**Table S1. Full feature list**

| **Section/Topic Item Development Checklist item**  **/ evaluation**^1^ | | | | **Reported on page** |
| --- | --- | --- | --- | --- |
| **TITLE** | | | |  |
| *Title* | 1 | D;E | Identify the study as developing or evaluating the performance of a multivariable prediction model, the target population, and the outcome to be predicted | √ |
| **ABSTRACT** | | | | |
| *Abstract* | 2 | D;E | See TRIPOD+AI for Abstracts checklist | √ |
| **INTRODUCTION** | | | | |
| *Background* | 3a | D;E | Explain the healthcare context (including whether diagnostic or prognostic) and rationale for developing or evaluating the prediction model, including references to existing models | √ |
|  | 3b | D;E | Describe the target population and the intended purpose of the prediction model in the context of the care pathway, including its intended users (e.g., healthcare professionals, patients, public) | √ |
|  | 3c | D;E | Describe any known health inequalities between sociodemographic groups | √ |
| *Objectives* | 4 | D;E | Specify the study objectives, including whether the study describes the development or validation of a prediction model (or both) | √ |
| **METHODS** | | | | |
| *Data* | 5a | D;E | Describe the sources of data separately for the development and evaluation datasets (e.g., randomised trial, cohort, routine care or registry data), the rationale for using these data, and representativeness of the data | √ |
|  | 5b | D;E | Specify the dates of the collected participant data, including start and end of participant accrual; and, if applicable, end of follow-up | √ |
| *Participants* | 6a | D;E | Specify key elements of the study setting (e.g., primary care, secondary care, general population)  including the number and location of centres | √ |
|  | 6b | D;E | Describe the eligibility criteria for study participants | √ |
|  | 6c | D;E | Give details of any treatments received, and how they were handled during model development or evaluation, if relevant | √ |
| *Data preparation* | 7 | D;E | Describe any data pre-processing and quality checking, including whether this was similar across  relevant sociodemographic groups | √ |
| *Outcome* | 8a | D;E | Clearly define the outcome that is being predicted and the time horizon, including how and when assessed, the rationale for choosing this outcome, and whether the method of outcome assessment is  consistent across sociodemographic groups | √ |
|  | 8b | D;E | If outcome assessment requires subjective interpretation, describe the qualifications and demographic characteristics of the outcome assessors | √ |
|  | 8c | D;E | Report any actions to blind assessment of the outcome to be predicted | √ |
| *Predictors* | 9a | D | Describe the choice of initial predictors (e.g., literature, previous models, all available predictors) and  any pre-selection of predictors before model building | √ |
|  | 9b | D;E | Clearly define all predictors, including how and when they were measured (and any actions to blind assessment of predictors for the outcome and other predictors) | √ |
|  | 9c | D;E | If predictor measurement requires subjective interpretation, describe the qualifications and demographic characteristics of the predictor assessors | √ |
| *Sample size* | 10 | D;E | Explain how the study size was arrived at (separately for development and evaluation), and justify that  the study size was sufficient to answer the research question. Include details of any sample size calculation | √ |
| *Missing data* | 11 | D;E | Describe how missing data were handled. Provide reasons for omitting any data | √ |
| *Analytical methods* | 12a | D | Describe how the data were used (e.g., for development and evaluation of model performance) in the analysis, including whether the data were partitioned, considering any sample size requirements | √ |
|  | 12b | D | Depending on the type of model, describe how predictors were handled in the analyses (functional form,  rescaling, transformation, or any standardisation). | √ |
|  | 12c | D | Specify the type of model, rationale^2^, all model-building steps, including any hyperparameter tuning,  and method for internal validation | √ |
|  | 12d | D;E | Describe if and how any heterogeneity in estimates of model parameter values and model performance was handled and quantified across clusters (e.g., hospitals, countries). See TRIPOD-Cluster for  additional considerations^3^ | √ |
|  | 12e | D;E | Specify all measures and plots used (and their rationale) to evaluate model performance (e.g., discrimination, calibration, clinical utility) and, if relevant, to compare multiple models | √ |
|  | 12f | E | Describe any model updating (e.g., recalibration) arising from the model evaluation, either overall or for particular sociodemographic groups or settings | √ |
|  | 12g | E | For model evaluation, describe how the model predictions were calculated (e.g., formula, code, object, application programming interface) | √ |
| *Class imbalance* | 13 | D;E | If class imbalance methods were used, state why and how this was done, and any subsequent methods to  recalibrate the model or the model predictions | √ |
| *Fairness* | 14 | D;E | Describe any approaches that were used to address model fairness and their rationale | √ |
| *Model output* | 15 | D | Specify the output of the prediction model (e.g., probabilities, classification). Provide details and  rationale for any classification and how the thresholds were identified | √ |
| *Training versus*  *evaluation* | 16 | D;E | Identify any differences between the development and evaluation data in healthcare setting, eligibility  criteria, outcome, and predictors | √ |
| *Ethical approval* | 17 | D;E | Name the institutional research board or ethics committee that approved the study and describe the participant-informed consent or the ethics committee waiver of informed consent | √ |
| **OPEN SCIENCE** | | | | |
| *Funding* | 18a | D;E | Give the source of funding and the role of the funders for the present study | √ |
| *Conflicts of interest* | 18b | D;E | Declare any conflicts of interest and financial disclosures for all authors | √ |
| *Protocol* | 18c | D;E | Indicate where the study protocol can be accessed or state that a protocol was not prepared | √ |
| *Registration* | 18d | D;E | Provide registration information for the study, including register name and registration number, or state  that the study was not registered | √ |
| *Data sharing* | 18e | D;E | Provide details of the availability of the study data | √ |
| *Code sharing* | 18f | D;E | Provide details of the availability of the analytical code^4^ | √ |
| **PATIENT & PUBLIC INVOLVEMENT** | | | | |
| *Patient & Public Involvement* | 19 | D;E | Provide details of any patient and public involvement during the design, conduct, reporting, interpretation, or dissemination of the study or state no involvement. | √ |
| **RESULTS** | | | | |
| *Participants* | 20a | D;E | Describe the flow of participants through the study, including the number of participants with and without the outcome and, if applicable, a summary of the follow-up time. A diagram may be helpful. | √ |
|  | 20b | D;E | Report the characteristics overall and, where applicable, for each data source or setting, including the key dates, key predictors (including demographics), treatments received, sample size, number of outcome events, follow-up time, and amount of missing data. A table may be helpful. Report any  differences across key demographic groups. | √ |
|  | 20c | E | For model evaluation, show a comparison with the development data of the distribution of important predictors (demographics, predictors, and outcome). | √ |
| *Model development* | 21 | D;E | Specify the number of participants and outcome events in each analysis (e.g., for model development, hyperparameter tuning, model evaluation) | √ |
| *Model specification* | 22 | D | Provide details of the full prediction model (e.g., formula, code, object, application programming interface) to allow predictions in new individuals and to enable third-party evaluation and implementation, including any restrictions to access or re-use (e.g., freely available, proprietary)^5^ | √ |
| *Model performance* | 23a | D;E | Report model performance estimates with confidence intervals, including for any key subgroups (e.g., sociodemographic). Consider plots to aid presentation. | √ |
|  | 23b | D;E | If examined, report results of any heterogeneity in model performance across clusters. See TRIPOD  Cluster for additional details^3^. | √ |
| *Model updating* | 24 | E | Report the results from any model updating, including the updated model and subsequent performance | √ |
| **DISCUSSION** | | | | |
| *Interpretation* | 25 | D;E | Give an overall interpretation of the main results, including issues of fairness in the context of the  objectives and previous studies | √ |
| *Limitations* | 26 | D;E | Discuss any limitations of the study (such as a non-representative sample, sample size, overfitting, missing data) and their effects on any biases, statistical uncertainty, and generalizability | √ |
| *Usability of the model in the context of current care* | 27a | D | Describe how poor quality or unavailable input data (e.g., predictor values) should be assessed and handled when implementing the prediction model | √ |
|  | 27b | D | Specify whether users will be required to interact in the handling of the input data or use of the model,  and what level of expertise is required of users | √ |
|  | 27c | D;E | Discuss any next steps for future research, with a specific view to applicability and generalizability of  the model | √ |

**Table S2**. **TRIPOD AI checklist**

|  | Established schizophrenia | | | First episode psychosis | |
| --- | --- | --- | --- | --- | --- |
|  | **Discovery** | **Internal Validation** | **External Validation** | **Discovery** | **External Validation** |
| **Total symptom severity** | r=0.68  (0.63-0.72) | r=0.75  (0.64-0.83) | r=0.40  (0.31-0.49) | r=0.44  (0.35-0.53) | r=0.5  (0.52-0.63) |
|  | <0.001 | <0.001 | <0.001 | <0.001 | <0.001 |
| **Percentage change in symptom severity** | r=0.15  (0.07-0.23) | r=0.1  (-0.11-0.31) | r=0.2  (0.09-0.3) | r=0.23  (0.13-0.34) | r=0.14  (0.07-0.22) |
|  | <0.001 | 0.35 | <0.001 | <0.001 | <0.001 |

.**Table S3**. **Model performances across the linear treatment outcomes for:**

**Left**: Established schizophrenia discovery sample, and **Right**: first episode psychosis discovery sample. Model performances are given in Pearson’s r for linear outcomes and Balanced Accuracy (BAC) for classification. Internal validation refers to different sites in the established schizophrenia. External validation columns refer to validation in the other sample. Confidence intervals are shown in brackets

|  | Established schizophrenia | | | | First episode psychosis | |
| --- | --- | --- | --- | --- | --- | --- |
|  |  | **Discovery** | **Internal Validation** | **External Validation** | **Discovery** | **External Validation** |
| **RSWG remission** | **Balanced Accuracy** | 69.0%  (68.4- 69.5) | 72.6%  (72.4-72.8) | 63.5%  (63.3-63.7) | 62.4%  (61.0-64.0) | 65.7%  (65.5-65.9) |
|  | **Sensitivity** | 63.9%  (63.6=64.7) | 56.0%  (53.2, 57.7) | 53.9%  (51.4.4-56.0) | 58.4%  (57.1-59.7) | 69.5%  (65.6-73.5) |
|  | **Specificity** | 74.0%  (73.1-75.3) | 87.9%  (87.4-89.3) | 73.1%  (71.1-75.7) | 66.3%  (64.9-68.3) | 61.9%  (57.5-65.0) |
|  | **Area Under the Curve** | 0.76  (0.72-0.80) | 0.81  (0.72-0.9) | 0.66  (0.60-0.73) | 0.68  (0.62-0.74) | 0.71  (0.66-0.76) |
|  | **p-value** | <0.001 | <0.001 | <0.001 | <0.001 | <0.001 |
| **25% symptom severity reduction** | **Balanced Accuracy** | 53.1%  (52.0-54.3) | 50.5%  (50.2-50.7) | 58.4%  (58.3-58.5) | 60.5%  (59.2-61.8) | 51.8%  (51.8-51.9) |
|  | **Sensitivity** | 54.6%  (53.5-56.0%) | 31.8%  (30.5-32.7) | 84.1%  (82.2-85,1) | 54.2%  (53.5-55.2) | 62.1%  (54.2-66.6) |
|  | **Specificity** | 56.2%  (53.9-57.2) | 66.7%  (65.4-67.4) | 42.6%  (39.8-44.0) | 67.4%  (63.6-69.7) | 41.9%  (40.9-52.7) |
|  | **Area Under the Curve** | 0.55  (0.51-0.6) | 0.49  (0.36-0.63) | 0.66  (0.57-0.75) | 0.67  (0.59-0.74) | 0.54  (0.41-0.51) |
|  | **p-value** | <0.001 | >0.99 | <0.001 | <0.001 | >0.99 |

**Table S4**. **Model performances across the binary treatment outcomes for:**

**Left**: Established schizophrenia discovery sample, and **Right**: first episode psychosis discovery sample. Model performances are given in Pearson’s r for linear outcomes and Balanced Accuracy (BAC) for classification. Internal validation refers to different sites in the established schizophrenia. External validation columns refer to validation in the other sample. Confidence intervals are shown in brackets

|  | Established schizophrenia | First episode psychosis |
| --- | --- | --- |
| Total symptom severity (mean (SD)) | 65.3 (17.2) | 49.8 (16.3) |
| RWSG remission (% remission) | 34.3% | 75.2% |

**Table S5. Distribution of the PANSS total and RSWG criteria at 12 months.**

**Left:** Established schizophrenia discovery sample, and **Right:** first episode psychosis sample.

PANSS total distribution is given in mean (standard deviation). The RWSG remission is represented as the percentage of participants in remission.

|  | Established schizophrenia | First episode psychosis |
| --- | --- | --- |
| Total symptom severity | r=0.53 | r=0.26 |
| RWSG remission | BAC=66.3% | BAC=57.1% |

**Table S6. Performance of the PANSS total models and RSWG models in predicting 12-month outcome.**

**Left:** Established schizophrenia discovery sample, and **Right:** First Episode Psychosis.

The models for predicting PANSS total and RSWG remission at 3 months were used to predict the same outcomes at 12 months. Model performances are given in Pearson’s r for linear outcomes and Balanced Accuracy (BAC) for classification.

|  |  | Female | Male | p-value |
| --- | --- | --- | --- | --- |
| Established schizophrenia | False non-remission (%) | 29.5% | 21.5% | 0.23 |
|  | False remission (%) | 70.5% | 78.5% |  |
| First episode psychosis | False non-remission (%) | 73.2% | 56.9% | 0.068 |
|  | False remission (%) | 26.8% | 43.1% |  |

**Table S7. Comparison of the number of participants who were falsely predicted as either remission or non-remission across sex groups**

P values were obtained via a Mann-Whitney test for the predicted scores, difference between expected and observed, and decision scores, and using a chi-squared test for the model misclassifications.

|  | | White | Non-White | P value |
| --- | --- | --- | --- | --- |
| Subgroup sample size | | n=384 (64.6%) | n=210 (35.4%) | N/A |
| Total symptom severity | **Median predicted scores** | 26.52 | 26.6 | **0.001** |
|  | **Median difference between observed and predicted scores** | 39.6 | 41.4 | 0.70 |
| RSWG remission | **Median decision scores** | 0.23 | 0.34 | **0.01** |
|  | **Model misclassifications** | 32.0% | 31.0% | 0.78 |

**Table S8. Performance of the external validation of the models developed in the first episode psychosis cohort in the established schizophrenia sample in subgroups with White and non-White ethnicity.**

P values were obtained via a Mann-Whitney test for the predicted scores, difference between expected and observed, and decision scores, and using a chi-squared test for the model misclassifications.

|  | Quetiapine | Ziprasidone | Olanzapine | Risperidone | Perphenazine | p-value |
| --- | --- | --- | --- | --- | --- | --- |
| False non-remission (%) | 72.2% | 81.0% | 16.7% | 65.6% | 67.6% | 0.48 |
| False remission (%) | 27.8% | 19% | 83.3% | 34.4% | 32.4% |  |

**Table S9**. **Comparison of the number of participants who were falsely predicted as either remission or non-remission across antipsychotic groups in the established schizophrenia sample:**

False non-remission: predicted by the model as being in non-remission when they actually remitted.

False remission: predicted by the model as being in remission when they actually did not remit.

Groups were compared using a Chi-squared test

|  | Quetiapine | Ziprasidone | Olanzapine | Amisulpride | Haloperidol | p-value |
| --- | --- | --- | --- | --- | --- | --- |
| False non-remission (%) | 60.9% | 70% | 72.4% | 52.2% | 68.4% | 0.58 |
| False remission (%) | 39.1% | 30% | 27.6% | 47.8% | 31.6% |  |

**Table S10**. **Comparison of the number of participants who were falsely predicted as either remission or non-remission across antipsychotic groups in the FEP sample:**

False non-remission: predicted by the model as being in non-remission when they actually remitted.

False remission: predicted by the model as being in remission when they actually did not remit.

Groups were compared using a Chi-squared test.

|  | | Quartile 1 | Quartile 2 | Quartile 3 | Quartile 4 | P value |
| --- | --- | --- | --- | --- | --- | --- |
| Established schizophrenia | **Total symptom severity** | r_z_=0.59 | r_z_=0.16 | r_z_=0.23 | r_z_=0.52 | 1.4x10^-61^ |
|  | **RWSG remission (% remission)** | BAC=57.1% | BAC=60.0% | BAC=62.3% | BAC=50.0% | 0.0008 |
| First episode psychosis | **Total symptom severity** | r_z_=0.33 | r_z_=0.13 | r_z_=0.14 | r_z_=0.29 | 1.25x10^-20^ |
|  | **RWSG remission (% remission)** | BAC=66.7% | BAC=58.3% | BAC=68.7% | BAC=53.6% | 6.1x10^-6^ |

**Table S11. Performance of the RSWG and total symptom severity models across the four quartiles of symptom severity.**

The median model performance across the 100 outer-fold partitions reported in Balanced Accuracy for the RSWG model and z-adjusted Pearson’s r for total symptom severity to account for the effect of subgroups. P-values were derived using a Kruskall-Wallis test on the model performances across the 100 outer folds. Pairwise comparisons are shown in **Figures S12 and S13**.

**References**

1. T. S. Stroup *et al.*, The National Institute of Mental Health Clinical Antipsychotic Trials of Intervention Effectiveness (CATIE) project: schizophrenia trial design and protocol development. *Schizophrenia bulletin* **29**, 15-31 (2003).

2. W. W. Fleischhacker, I. P. Keet, R. S. Kahn, The European First Episode Schizophrenia Trial (EUFEST): rationale and design of the trial. *Schizophr Res* **78**, 147-156 (2005).

3. D. V. Sheehan *et al.*, The Mini-International Neuropsychiatric Interview (M.I.N.I.): the development and validation of a structured diagnostic psychiatric interview for DSM-IV and ICD-10. *The Journal of clinical psychiatry* **59 Suppl 20**, 22-33;quiz 34-57 (1998).

4. B. First M, Structured Clinical Interview for DSM-IV Axis I Disorders. *Biometrics Research Department*, (1997).

5. C. M. Adler *et al.*, Neurochemical effects of quetiapine in patients with bipolar mania: A proton magnetic resonance spectroscopy study. *Journal of Clinical Psychopharmacology* **33**, 528-532 (2013).
